# Mining the Methylome Reveals Extensive Diversity in Staphylococcus epidermidis Restriction Modification

**DOI:** 10.1128/mBio.02451-19

**Published:** 2019-12-17

**Authors:** Jean Y. H. Lee, Glen P. Carter, Sacha J. Pidot, Romain Guérillot, Torsten Seemann, Anders Gonçalves da Silva, Timothy J. Foster, Benjamin P. Howden, Timothy P. Stinear, Ian R. Monk

**Affiliations:** a Department of Microbiology and Immunology, The University of Melbourne at The Doherty Institute for Infection and Immunity, Victoria, Australia; b Microbiological Diagnostic Unit Public Health Laboratory, Department of Microbiology and Immunology, The University of Melbourne at The Doherty Institute for Infection and Immunity, Victoria, Australia; c Doherty Applied Microbial Genomics, Department of Microbiology and Immunology, The University of Melbourne at The Doherty Institute for Infection and Immunity, Victoria, Australia; d Moyne Institute of Preventative Medicine, Department of Microbiology, School of Genetics and Microbiology, Trinity College Dublin, Dublin, Ireland; e Infectious Diseases Department, Austin Health, Victoria, Australia; New York University School of Medicine

**Keywords:** DNA methylation, *Staphylococcus aureus*, coagulase-negative staphylococci, generalized transduction, genetic manipulation, genome analysis, type I restriction modification

## Abstract

Staphylococcus epidermidis is a significant opportunistic pathogen of humans. Molecular studies in this species have been hampered by the presence of restriction-modification (RM) systems that limit introduction of foreign DNA. Here, we establish the complete genomes and methylomes for seven clinically significant, genetically diverse S. epidermidis isolates and perform the first systematic genomic analyses of the type I RM systems within both S. epidermidis and Staphylococcus aureus. Our analyses revealed marked differences in the gene arrangement, chromosomal location, and movement of type I RM systems between the two species. Unlike S. aureus, S. epidermidis type I RM systems demonstrate extensive diversity even within a single genetic lineage. This is contrary to current assumptions and has important implications for approaching the genetic manipulation of S. epidermidis. Using Escherichia coli plasmid artificial modification (PAM) to express S. epidermidis *hsdMS*, we readily overcame restriction barriers in S. epidermidis and achieved electroporation efficiencies equivalent to those of modification-deficient mutants. With these functional experiments, we demonstrated how genomic data can be used to predict both the functionality of type I RM systems and the potential for a strain to be electroporation proficient. We outline an efficient approach for the genetic manipulation of S. epidermidis strains from diverse genetic backgrounds, including those that have hitherto been intractable. Additionally, we identified S. epidermidis BPH0736, a naturally restriction-defective, clinically significant, multidrug-resistant ST2 isolate, as an ideal candidate for molecular studies.

## Abstract (precis)

Staphylococcus epidermidis is a major cause of hospital-acquired infections, especially those related to implanted medical devices. Understanding how S. epidermidis causes disease and devising ways to combat these infections have been hindered by an inability to genetically manipulate clinically significant hospital-adapted strains. Here, we provide the first comprehensive analyses of the barriers to the uptake of foreign DNA in S. epidermidis and demonstrate that these are distinct from those described for S. aureus. Using these insights, we demonstrate an efficient approach for the genetic manipulation of S. epidermidis to enable the study of clinical isolates for the first time.

## INTRODUCTION

Staphylococcus epidermidis is a ubiquitous colonizer of human skin (1). Invasive medical procedures, specifically, insertion of prosthetic devices on which the bacteria can form a biofilm, enable evasion of both antibiotics and the host immune system, which has contributed to its increasing importance as a significant nosocomial pathogen. A leading cause of surgical-site- and central-line-associated bloodstream infections (2), S. epidermidis poses a major economic burden (3). In the hospital environment, two multilocus sequence types (MLSTs), ST2 and ST23, account for most clinical disease (4, 5). Three hospital-adapted clones (two ST2 and one ST23) were recently demonstrated to be globally disseminated and to have evolved to become untreatable with first-line agents though the acquisition of multiple antibiotic resistance determinants and resistance-conferring mutations (5). Efforts to increase knowledge concerning the molecular genetics, pathogenesis, and treatment of S. epidermidis have been limited by barriers preventing the genetic manipulation of clinically relevant isolates and the assumption that the characteristics of S. epidermidis are similar to those of S. aureus.

Restriction-modification (RM) systems have evolved as a form of bacterial immunity that degrades incoming DNA from foreign donors such as bacteriophage (6). Type I and IV RM systems represent a significant barrier to genetic manipulation of staphylococci. Type I RM systems are comprised of three host specificity for DNA (*hsd*) genes that encode (i) a specificity protein (HsdS), (ii) a modification protein (HsdM), and (iii) a restriction endonuclease (HsdR). Together, these function as a single protein complex in which HsdS determines the DNA target recognition motif (TRM) in which adenine residues are methylated by HsdM, while HsdR cleaves unmodified and non-self-modified DNA (7, 8). Type IV RM systems consist of a single restriction endonuclease that cleaves DNA with inappropriate modification (8).

Plasmid artificial modification (PAM) is a method to overcome the barrier imposed by RM systems where plasmid DNA is passaged through a cytosine methylation-deficient Escherichia coli host (DC10B) that has been engineered to heterologously express the *hsdMS* system of the staphylococcal strain to be transformed. Plasmid DNA extracted from this E. coli host mimics the DNA methylation profile of the target strain, thus enabling introduction of plasmid DNA and subsequent genetic manipulation (9).

Type I RM systems of staphylococci are best understood in S. aureus. The distribution of *hsdS* alleles corresponds to clonal complex (CC) for the 10 dominant S. aureus lineages (10). Far less is known about the type I RM systems in S. epidermidis. A recent study suggested that S. epidermidis type I RM systems adhered to lineage-specific groupings like S. aureus. However, this inference was based on analysis of only four new S. epidermidis methylomes (11) plus the one methylome that had already been characterized; namely, the ST2 reference genome of strain BPH0662 (12).

Here, we present the first systematic genomic analyses of the type I RM systems in S. aureus and S. epidermidis and demonstrate how these data can be used to predict functionality of type I RM systems and associated competence of strains. We show that PAM is a highly efficient method to enable genetic manipulation of S. epidermidis, particularly hospital-adapted isolates that possess multiple functional type I RM systems.

## RESULTS AND DISCUSSION

### S. aureus type I RM systems are lineage specific.

**FIG 1:**
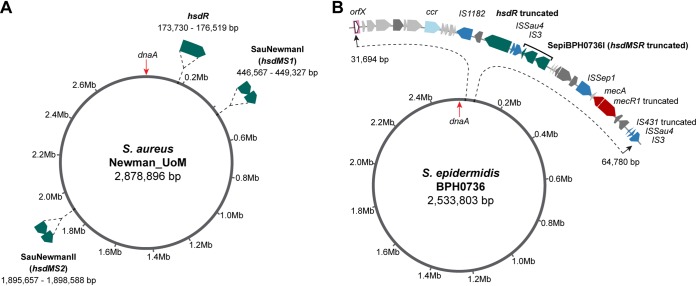
Comparison of the structures and chromosomal locations of S. aureus and S. epidermidis type I restriction-modification systems. (A) S. aureus Newman_UoM (29, 32). (B) S. epidermidis BPH0736. For consistency, the chromosome is orientated forwards starting at the start codon of *dnaA*, and native type I RM systems are sequentially numbered.

**FIG 2:**
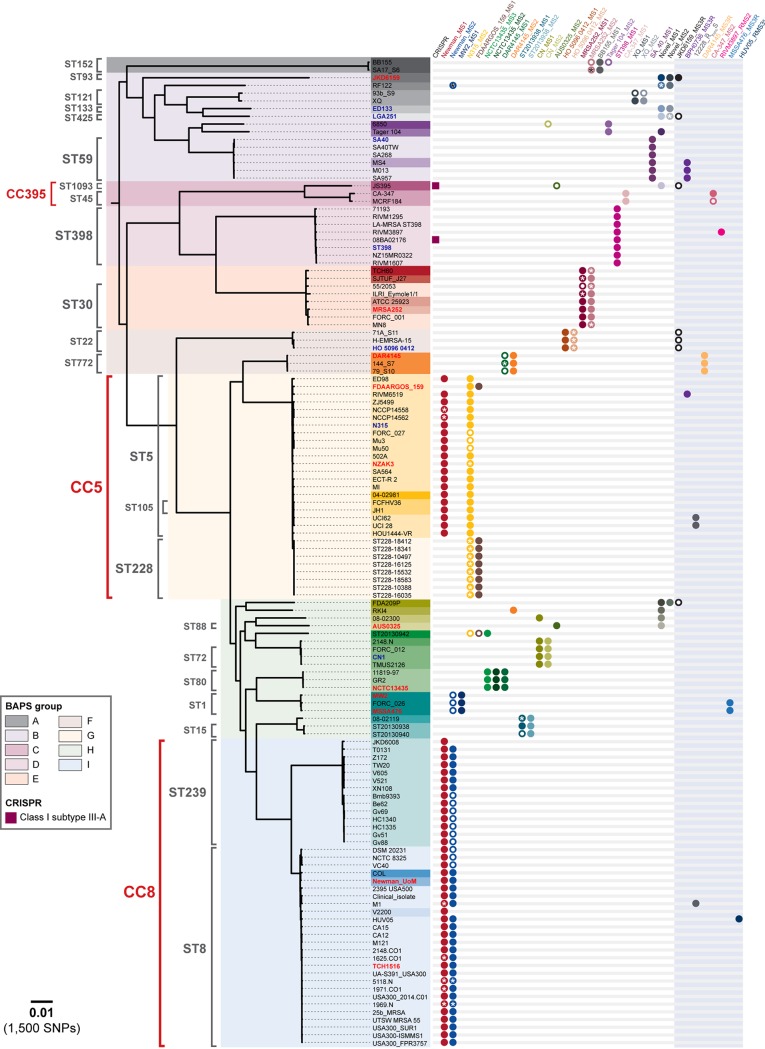
S. aureus native type I restriction-modification systems are lineage specific. The figure shows a maximum likelihood core SNP-based phylogeny of 128 closed S. aureus genomes originating from 40 STs, using Newman_UoM as the reference genome. Overlaid are the results of *in silico* multilocus sequence type (MLST), clonal cluster (CC), Bayesian analysis of population structure (BAPS), presence of CRISPR-Cas systems, and type I restriction-modification system HsdS variants. Bold red font indicates isolates with PacBio-characterized methylomes. Bold blue font indicates isolates with methylomes determined by DNA cleavage with purified enzyme. Boxes around strain names are colored according ST type. Open circles represent amino acid substitutions present in HsdS. An asterisk (*) indicates a truncated HsdS subunit. The scale bar indicates the number of nucleotide substitutions per site (bold) with an approximation of SNP rate (in parentheses).

We began this study by testing the notion that S. aureus type I RM systems are lineage specific. We compared 128 publicly available finished S. aureus genome sequences (see Table S1A in the supplemental material) and confirmed that the chromosomal location and structure of type I RM systems in S. aureus are highly conserved. A total of 110 genomes had a single *hsdR* gene and two copies of *hsdMS* with the first in forward orientation located in the alpha pathogenicity island and the second in the opposite orientation and located within the beta pathogenicity island (10, 13) (Fig. 1A). The remaining 18 strains possessed *hsdR* and a single copy of forward oriented *hsdMS* in the alpha pathogenicity island. Five of the 128 strains possessed a third type I RM system at a nonmobile chromosomal location downstream of *lacA*. Twenty-three strains carried an additional type I RM system on a mobile genetic element, 22 were on staphylococcal cassette chromosome (SCC) elements (Fig. 2; see also Fig. S1 in the supplemental material), and 1 carried a type I RM system on a plasmid (strain HUV05). Single variants of both HsdR (NCBI protein accession no. WP_000331347.1; *n* = 127) and HsdM (WP_000028628.1; *n* = 222) were demonstrated for the type I RM systems situated in nonmobile chromosomal locations, indicating stable vertical inheritance. Interruptions in *hsdR* and *hsdMS* due to horizontal gene transfer were rarely seen in S. aureus. A solitary example of *hsdS* truncation due to insertion of a bacteriophage was noted in strain Sa17_S6. Most changes were due to single nucleotide polymorphisms (SNPs) leading to amino acid substitutions (*n* = 35) or nonsense mutations (*n* = 31) in *hsdS* (Fig. 2). See Table 1 for a comparison of S. aureus and S. epidermidis type I RM systems.

**TABLE 1:** Comparison of S. aureus and S. epidermidis type I restriction-modification systems

Staphylococcus aureus type I RM system characteristics	Staphylococcus epidermidis type I RM system characteristics
RM system organized as a single *hsdR* gene separated from one, two, or three distant *hsdMS* gene pairs	RM system organized as complete three-gene operon (*hsdRMS* or *hsdMSR*)
Conserved, stable chromosomal location for each gene	Close proximity to *ccr* genes integrated at *orfX*, located in a highly plastic region of the genome
Most strains have two type I RM systems	Most strains have a single type I RM system
All strains have at least one type I RM system	Many (38.1%) strains have no type I RM system
Up to three functional type I RM systems per isolate	Up to three functional type I RM systems per isolate
99.7% amino acid pairwise identity for all native HsdRs	At least five identified variants of HsdR
99.3% amino acid pairwise identity for all native HsdMs	At least six identified variants of HsdM
At least 48 different variants of HsdS (8 likely imported from coagulase-negative staphylococci)	At least 31 different variants of HsdS
Relative conservation of HsdS present within ST groups	No clear conservation of HsdS according to ST group
Conservation of HsdM provides redundancy, enabling interaction with multiple different HsdS	Each HsdS is capable of interacting only with the corresponding paired HsdM; therefore, not all orphan *hsdS* genes are functional
Complete three-gene *hsdRMS*/*MSR* type I systems carried on SCC elements do not adhere to lineage specificity and are likely imported from coagulase-negative staphylococci	

10.1128/mBio.02451-19.2 FIG S1 A hypothesized role for cassette chromosome recombinase (*ccr*) in the mobilization of S. epidermidis and S. aureus type I restriction-modification systems. (A) Complete S. epidermidis genomes with type I RM systems. (B) S. aureus genomes with imported type I RM systems. The genomes are orientated forwards starting at *dnaA*. A number sign (#) indicates an NCBI uploaded genome that does not start at *dnaA*. An asterisk (*) indicates a strain not classifiable by existing MLST scheme. Download FIG S1, PDF file, 1.6 MB. Copyright © 2019 Lee et al. 2019 Lee et al. This content is distributed under the terms of the Creative Commons Attribution 4.0 International license.

10.1128/mBio.02451-19.5 TABLE S1 Metadata associated with preexisting S. aureus and S. epidermidis sequencing. (A) S. aureus reference genomes. (B) S. epidermidis reference genomes. (C) S. epidermidis SRA isolate metadata. (D) Isolate metadata from Lee et al. (5). (E) Isolate metadata from Costa et al. (11). Download TABLE S1, XLSX file, 0.1 MB. Copyright © 2019 Lee et al. 2019 Lee et al. This content is distributed under the terms of the Creative Commons Attribution 4.0 International license.

We next established a high-resolution phylogeny using 144,727 core genome SNPs for the 128 S. aureus genomes covering 40 STs (Fig. 2). Occurrences of *hsdMS* genes were mapped across the phylogeny for each genome. A total of 48 HsdS subunits were identified with associated TRMs (Table 2; see also Table S2A [https://melbourne.figshare.com/articles/Sa_HsdS_48_fasta/7986956]). Although the same *hsdMS* genes were present in genetically distinct lineages, the combinations of *hsdMS* genes were conserved within each lineage (Fig. 2). For example, the same two HsdMS products were present in ST250 and ST254, which are single-locus variants of ST8. A notable exception to the lineage specificity was represented by the type I RM systems carried on SCC elements (Fig. S1B), which may have been acquired from coagulase-negative staphylococci (CoNS). Complete S. epidermidis BPH0736 *hsdMSR* genes (nondisrupted, identical sequences) were observed in four S. aureus strains from three different STs (ST5, ST59, and ST338), suggesting gene transfer between the species (Fig. S1).

**TABLE 2:**
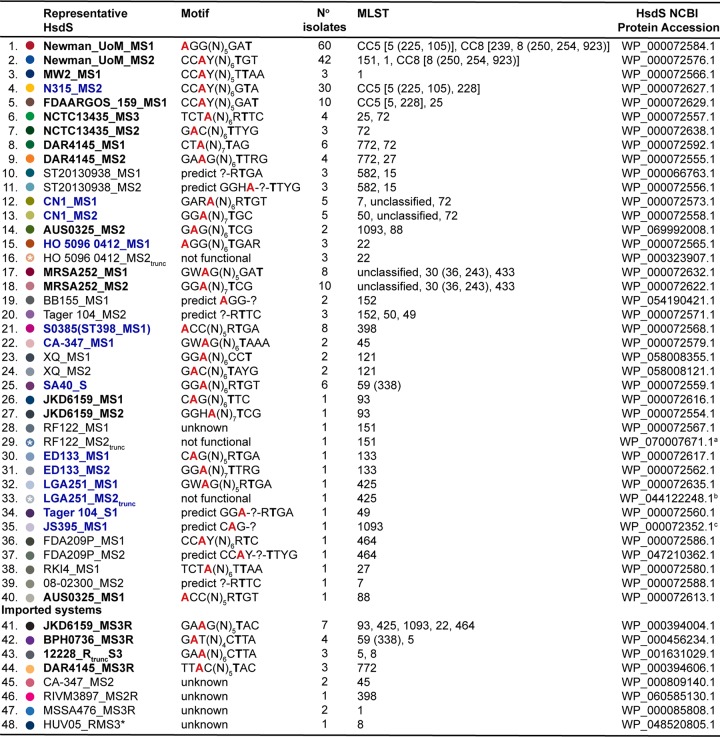
Diversity of S. aureus type I restriction-modification system methylation profiles^*d*^

a Truncation at amino acid 203.

b Truncation at amino acid 249.

c Truncation at amino acid 8. Full amino acid translations of all 48 HsdS variants are accessible at Figshare (https://melbourne.figshare.com/articles/Sa_HsdS_48_fasta/7986956).

d Isolate HsdS motifs were collated from publications by Monk et al. (9) and Cooper et al. (18) and from the REBASE database (33). HsdS names in bold black font have motifs determined by PacBio sequencing of the isolate after which the representative HsdS was named. HsdS names in bold blue font have motifs determined by DNA cleavage with purified restriction enzyme. The multilocus sequence types (MLSTs) in which each HsdS was found are listed according to the order in which they appear in the Fig. 2 phylogeny (top to bottom). STs within the same clonal complex (CC) are listed within square brackets; STs within parentheses represent single-locus variants of the ST group they are listed after. trunc, truncated; A (red), methylated adenine residue; T, complementary partner to methylated adenine residue. *, HUV05_RMS3 is carried on a plasmid, not integrated in the chromosome.

10.1128/mBio.02451-19.6 TABLE S2 S. aureus and S. epidermidis type I restriction-modification system subunit protein accession numbers and references. (A) S. aureus HsdS. (B) S. aureus HsdR and HsdM. (C) S. epidermidis HsdS. (D) S. epidermidis HsdR and HsdM. Download TABLE S2, XLSX file, 0.04 MB. Copyright © 2019 Lee et al. 2019 Lee et al. This content is distributed under the terms of the Creative Commons Attribution 4.0 International license.

### S. epidermidis type I RM systems are carried on mobile genetic elements.

Seven complete S. epidermidis reference genomes were publicly available at the beginning of this study (Table S1). Of these, only BPH0662 (12) and RP62a had characterized type I RM system motifs. However, the RP62a methylome was determined independently (11) of the finished genome (14). The methylomes of S. epidermidis isolates 1457 (15) and 14.1.R1 (16) confirmed that, consistent with the absence of *hsdM* genes, neither possessed a functional type I RM system. To improve understanding of the type I RM systems in S. epidermidis, we conducted PacBio SMRT sequencing and established complete genomes and adenine methylomes for six additional S. epidermidis strains from ST2, ST5, ST59, and ST358 and we resequenced RP62a (ST10) (See Table S3 for metadata).

10.1128/mBio.02451-19.7 TABLE S3 S. epidermidis reference isolate metadata. (A) Clinical metadata. (B) Closed-genome statistics. (C) PacBio methylation statistics. (D) CRISPR. (E) Vitek 2 susceptibilities. (F) Resistome. (G) Susceptibilities to commonly used plasmid selection markers. Download TABLE S3, XLSX file, 0.03 MB. Copyright © 2019 Lee et al. 2019 Lee et al. This content is distributed under the terms of the Creative Commons Attribution 4.0 International license.

The typical chromosomal arrangement of the type I RM system in S. epidermidis is shown for BPH0736 (ST2) (Fig. 1B). Unlike S. aureus (Fig. 1A), type I RM systems in S. epidermidis are arranged as a complete three-gene operon in either an *hsdRMS* or *hsdMSR* organization, unless interrupted (Fig. S1). Analyses of the 11 closed S. epidermidis genomes containing type I RM systems demonstrated their co-occurrence with cassette chromosome recombinase (*ccr*) genes (with or without the presence of *mecA*). The 16 type I RM systems present within these 11 genomes were located a mean distance of 11.5 kb (minimum, 2.3 kb; maximum, 51.0 kb) from the nearest *ccr* (Fig. S1A). Similarly, in the 22 S. aureus genomes with a SCC-associated type I RM system, the mean distance between *hsdRMS*/and *hsdMSR* and the nearest *ccr* was 6.9 kb (minimum, 1.6 kb; maximum, 20.9 kb) (Fig. S1B).

Cassette chromosome recombinases typically integrate at *orfX* (corresponding to the last 15 nucleotides of the rRNA large subunit methyltransferase [17]), which is located at 31.6 kb in the S. epidermidis chromosome (33.3 kb in S. aureus). This represents the start of a highly plastic region of the chromosome, in which multiple antibiotic resistance genes and genes corresponding to drug transporters and insertion sequence (IS) elements have accumulated (12) (Fig. S1). All 11 S. epidermidis and 22 S. aureus genomes with *ccr-*associated type I RM systems were integrated at *orfX* (Fig. S1). For type I RM system variants present in multiple isolates, conservation of genes surrounding the system and *ccr* was observed, consistent with the mobilization of an entire element (Fig. S1). Preserved cassette structure between isolates and in both species (Fig. S1B) led us to hypothesize that the movement of type I RM systems in S. epidermidis is mediated by *ccr*, enabling mobilization on SCC elements between strains and to other staphylococcal species. Localization in this region of the genome also predisposes S. epidermidis type I RM systems to disruption, potentially rendering variants restriction deficient. This was seen with interruption of *hsdR* by IS elements in BPH0736 (Fig. 1B).

### S. epidermidis type I RM systems are not strictly conserved within lineages.

To expand the S. epidermidis data set, we added short-read data from 234 publicly available S. epidermidis genomes to the 13 finished genomes (Table S1). In contrast to the S. aureus data, variability was noted in both the HsdR and HsdM subunits for S. epidermidis. Across the 247 genomes, 183 intact HsdR genes were identified, including five major HsdR variants (<90% amino acid pairwise identity threshold) (Table S2 [https://melbourne.figshare.com/articles/Se_HsdR_5_fasta/7986893]). The two variants of HsdR in strain BPH0662 shared only 22% amino acid identity. Similarly, 178 complete HsdM genes were identified with six major variants (Table 3; see also Table S2) (https://melbourne.figshare.com/articles/Se_HsdM_6_fasta/7986827). The amino acid sequences of these six variants were markedly divergent. The two variants of HsdM present in BPH0662 shared only 31% amino acid identity.

**TABLE 3:**
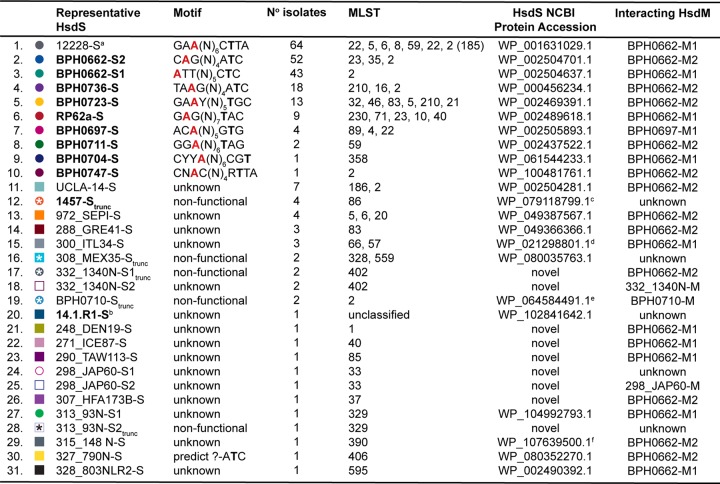
Diversity of S. epidermidis type I restriction modification methylation profiles^*g*^

a The ATCC 12228 type I RM system is nonfunctional, with a truncated *hsdR* gene, a complete *hsdS* gene, and no *hsdM* gene. All 64 isolates possessed the same incomplete type I RM system. The motif was identified based on the methylome determined for NIH4008 due to the presence of an HsdM protein capable of interacting with 12228 HsdS.

b 14.1.R1 type I RM system is nonfunctional, with truncated *hsdR*, complete *hsdS*, and no *hsdM.*

c L1M substitution.

d First 81 amino acids truncated.

e S295P substitution.

f Eleven amino acid substitutions (K26E, I56V, E59K, E171K, K174R, K175T, E178A, I193V, D201N, Y386F, and V434I). Amino acid translations of all 31 HsdS variants (https://melbourne.figshare.com/articles/Se_HsdS_31_fasta/7986911) and their interacting HsdMs (https://melbourne.figshare.com/articles/Se_HsdM_6_fasta/7986827) are accessible through Figshare.

g Isolate HsdS motifs were collated from methylomes newly characterized in this study and from publications by Lee et al. (12) and Costa et al. (11). HsdS names in bold black font have motifs determined by PacBio sequencing of the isolate after which the representative HsdS was named. The multilocus sequence types (MLSTs) in which each HsdS was found are listed according to the order in which they appear in the Fig. 3 phylogeny (clockwise). ST185 is a single-locus variant of ST2. trunc, truncated; A (red), methylated adenine residue; T, complementary partner to methylated adenine residue.

A maximum likelihood phylogeny for the 247 S. epidermidis genomes, derived from 83,210 core SNPs and sampled from 72 STs, was established, and the 31 different S. epidermidis HsdS subunits identified were overlaid (Fig. 3). Where known, their associated TRMs are shown in Table 3 with NCBI protein accession numbers (see also Table S2). Amino acid sequences of all 31 HsdS are available from Figshare (https://melbourne.figshare.com/articles/Se_HsdS_31_fasta/7986911). The distribution of S. epidermidis HsdS proteins within the population differed markedly from that observed within S. aureus, with no strict concordance to lineage specificity. For example, HsdS from BPH0723 (BPH0723-S; Table 3) was present in 13 isolates from five STs (ST5, ST21, ST46, ST210, and one unclassified ST), while BPH0662-S2 was identified in 52 isolates from three STs (ST2, ST23, and ST35). Although a high proportion of ST2 isolates shared the same predicted methylome (Fig. 3), the majority of these were known to be clones of internationally disseminated, multidrug-resistant strain BPH0662 (5). However, even within this highly clonal group (*n* = 36), some predicted methylation variation existed. For example, BPH0662-S2 was absent from two isolates, six isolates (including BPH0662) had a truncation in the 12228-S orphan system, and two isolates were missing the 12228-S orphan system completely. Furthermore, within ST2, seven different variants of HsdS were identified in 11 arrangements, including the absence of any type I RM system (Fig. 3). Of the 247 S. epidermidis genomes analyzed, 38% did not contain any *hsdS* alleles and were predicted to be restriction deficient.

**FIG 3:**
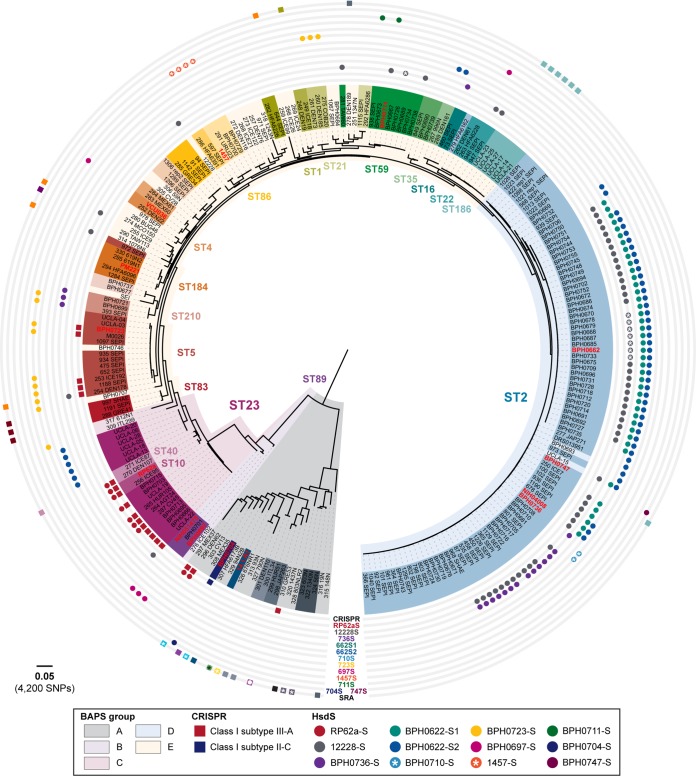
S. epidermidis type I restriction-modification systems are not conserved within lineages. The figure shows a maximum likelihood, core SNP-based phylogeny for 247 S. epidermidis genomes, including 7 newly closed reference genomes, 6 existing reference genomes, 156 genomes curated from the NCBI sequence read archive (SRA), 75 isolates from a study by Lee et al. (5); and the 3 draft genomes with methylation data (11). BPH0736 was used as the reference genome for analyses. Overlaid are the results of *in silico* multilocus sequence type (MLST), Bayesian analysis of population structure (BAPS), presence of CRISPR-Cas systems, and type I restriction-modification system HsdS variants. Bold red font indicates isolates with characterized methylomes. Isolates were from 70 recognized and two unclassified MLST groups. Boxes around strain names are colored according ST type; where background color is same as that of the BAPS group, the result indicates an ST represented by a single isolate. An asterisk (*) represents a truncated HsdS subunit. The scale bar indicates the number of nucleotide substitutions per site (bold) with an approximation of SNP rate (in parentheses).

Methylation analysis performed using our Pacbio-sequenced, Illumina read-corrected RP62a_UoM genome as a reference indicated the presence of a single type I RM system with a G**A**GN_7_TAC TRM (Table 3). Although this motif was consistent with that previously reported by Costa et al. (11), the three additional motifs previously described (lacking apparent associated genes) were not detected by our methylome analysis; the low complexity of the motifs (e.g., GGBNNH) and the low frequency of detected methylation (12% to 29%) (11) suggest that these might have represented artifacts rather than true motifs. Similarly, the three additional low-complexity and low-frequency motifs reported for VCU036 (11) probably represented artifacts. Although the ST type was not specified, VCU036, which shared the same methylome with ST89 isolate NIH051475, was reported as CC89 by Costa et al., leading to the conclusion that S. epidermidis type I RM systems follow S. aureus*-*like lineage specificity (11). We performed *in silico* MLST by two independent methods and determined that VCU036 belongs to ST4. Furthermore, our analysis of the 247 S. epidermidis genomes demonstrated VCU036 to be phylogenetically distinct from ST89 (Fig. 3).

Overall, our analyses demonstrated that, in contrast to current assumptions (11), the type I RM systems of S. epidermidis do not adhere to the lineage-specific distribution observed in S. aureus. These differences are attributable to the arrangement of S. epidermidis type I RM systems as complete three-gene operons that reside within a highly mobile region of the chromosome, the movement of which we hypothesize to be mediated by *ccr*.

### Recombinant target recognition domains generate HsdS variants with low conservation of amino acid identity.

The structure of a typical type I RM system HsdS allele is shown in Fig. S2A and is composed of two highly variable target recognition domains (TRDs) flanked and separated by conserved regions (CRs) that collectively determine the methylation of the TRM by HsdM. Recombinant pairings of TRDs result in different variants of HsdS (13, 18). Alignments of the range of S. aureus and S. epidermidis HsdS proteins identified in this study are shown in Fig. S2B and S3, respectively. Within our S. aureus and S. epidermidis collections, 77 variants of HsdS that shared only 24% pairwise identity were identified. This low level of conservation poses a potential challenge to the high-throughput bioinformatic screening for HsdS variants within genomic data sets. However, using HsdS from ATCC 12228 as the reference translation with our described method, we were able to detect the partial if not complete presence of all HsdS variants in both species. Of note, 12228-S was the only HsdS variant found within both species that clustered with the majority of S. aureus variants. In comparison, RP62a-S captured only 18 of the 31 S. epidermidis HsdS variants and fragments of fewer than half of the S. aureus HsdS variants.

10.1128/mBio.02451-19.3 FIG S2 Alignment of S. aureus HsdS variants. (A) Structure of an HsdS allele with conserved regions (CRs) flanking two variable regions known as target recognition domains (TRD1 and TRD2). (B) Each TRD typically specifies three to four defined base pairs, including a methylated adenine residue (red A; T, complementary partner to methylated adenine residue); with a four-to-seven-base-pair nonspecific spacer (N) between the two defined halves; collectively, these TRDs determine the full target recognition motif (TRM) specified by an HsdS variant. HsdS names in bold black font have motifs determined by PacBio sequencing of the isolate, after which the representative HsdS was named. HsdS names in bold blue font have motifs determined by DNA cleavage with a purified restriction enzyme. Alignments of the identified variants of S. aureus HsdS are shown adjacent to their TRMs, each formed by a different TRD pairing. The scale above the alignments indicates the position in the consensus alignment, with the mean pairwise identity at each site graphed (green, 100% identity; khaki, 30% to 100% identity; red, <30% identity). Blue (TRD1) and red (TRD2) outlines highlight examples of TRDs that recur within the alignments and the TRM base pairs that they define. Yellow boxes highlight alignments of HsdS imported into S. aureus on staphylococcal cassette chromosome elements. Download FIG S2, PDF file, 0.9 MB. Copyright © 2019 Lee et al. 2019 Lee et al. This content is distributed under the terms of the Creative Commons Attribution 4.0 International license.

10.1128/mBio.02451-19.4 FIG S3 Alignment of S. epidermidis HsdS variants. HsdS names in bold black font have motifs determined by PacBio sequencing of the isolate after which the representative HsdS was named. Target recognition motifs (TRMs) (when known) and their associated amino acid alignments are shown in adjacent positions. The scale above the alignments indicates the position in the consensus alignment, with mean pairwise identity at each site graphed (green, 100% identity; khaki, 30% to 100% identity; red, <30% identity). A red outline highlights examples of target recognition domains that recur within the alignments and the TRM base pairs that they define. A yellow box highlights the 12228-S alignment; this HsdS variant was found to be present in both S. aureus and S. epidermidis and shared conserved regions with the S. aureus HsdS variants located within stable chromosomal islands (Fig. S2). Download FIG S3, PDF file, 0.6 MB. Copyright © 2019 Lee et al. 2019 Lee et al. This content is distributed under the terms of the Creative Commons Attribution 4.0 International license.

### S. epidermidis HsdS variants interact only as part of a specific complex.

The arrangement of some S. epidermidis type I RM systems, with the presence of a truncated *hsdS* gene between complete *hsdR* and *hsdMS* genes, suggested the occurrence of recombination of component genes (e.g., S. epidermidis BPH0662I [SepiBPH0662I], SepiRP62aI, and SepiBPH0704I; Fig. S1). Analyses of the 247 genomes indicated that each variant of *hsdS* in S. epidermidis was always associated with a specific *hsdR* gene and *hsdM* gene, with a gene arrangement that was conserved (unless interrupted), frequently with the same surrounding genes found in association with *ccr* (Fig. S1A). These observations support our hypothesis of a role for SCC elements in the mobilization of S. epidermidis type I RM systems.

The presence of an orphan *hsdS* gene without a partner *hsdM* gene in S. epidermidis introduces additional complexity to the prediction of type I RM system functionality. This was demonstrated by the presence of 12228-S, the most prevalent HsdS within the data set, in 64 S. epidermidis isolates (Table 3) (Fig. 3) and three S. aureus isolates (Table 2 and Fig. 2). All examples of this *hsdS* variant followed a truncated *hsdR* gene, without an *hsdM* gene. We determined that 12228-S was expressed only when the corresponding specific interacting variant of *hsdM* (BPH0662-M1; WP_002504638.1) was also present (see Table S4 for full explanation). In contrast, conservation of a single variant of *hsdM* present twice within the same S. aureus genome provides redundancy for the expression of type I RM methylation. This is consistent with previous findings where the product of a single copy of the conserved S. aureus *hsdM* allele could functionally interact with both CC8 HsdS products when heterologously expressed in E. coli (9).

10.1128/mBio.02451-19.8 TABLE S4 Determining the HsdM variant that interacts with ATCC 12228 HsdS. Download TABLE S4, DOCX file, 0.02 MB. Copyright © 2019 Lee et al. 2019 Lee et al. This content is distributed under the terms of the Creative Commons Attribution 4.0 International license.

### Plasmid artificial modification to overcome the type I RM systems in S. epidermidis provides electroporation efficiency equivalent to deletion of functional type I systems.

**FIG 4:**
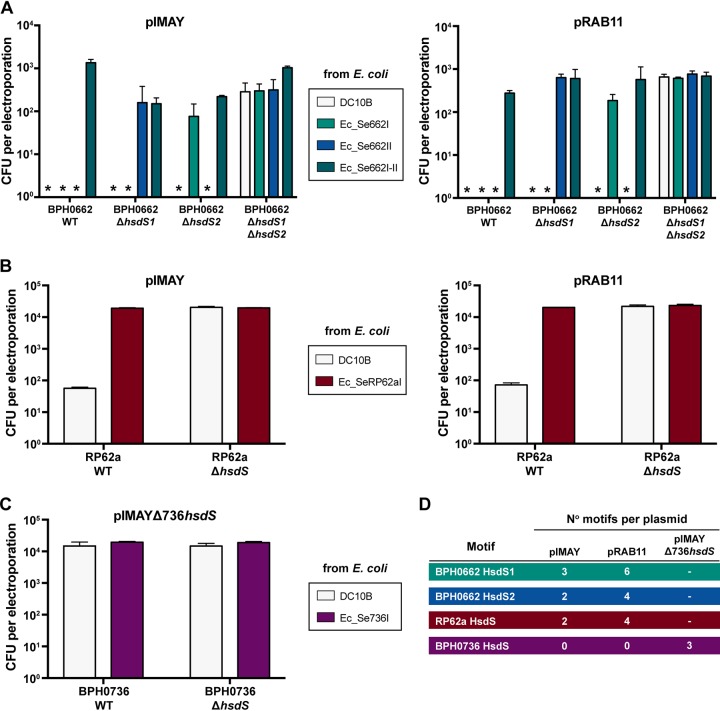
Plasmid artificial modification to overcome the type I RM systems in S. epidermidis. Biological triplicate data were determined for 5 μg of plasmid passaged through DC10B E. coli compared to the relevant E. coli PAM construct and transformed into S. epidermidis wild-type (WT) and Δ*hsdS* mutant strains. Error bars represent means ± standard deviations of results from three independent experiments. *, no transformants. (A) Electroporation of BPH0622-WT, BPH0662 Δ*hsdS1*, BPH0662 Δ*hsdS2*, and BPH0662 Δ*hsdS1* Δ*hsdS2* with plasmid pIMAY (left) or pRAB11 (right) isolated from DC10B and strain-specific E. coli Ec_Se662I (expressing BPH0662*hsdMS1*), Ec_Se662II (expressing BPH0662*hsdMS2*), and Ec_Se662I-II (expressing both BPH0662*hsdMS1* and BPH0662*hsdMS2*). (B) Electroporation of RP62a-WT and RP62a Δ*hsdS* with plasmid pIMAY (left) or pRAB11 (right) isolated from DC10B and strain-specific E. coli Ec_SeRP62aI (expressing RP62a*hsdMS*). (C) Electroporation of BPH0736-WT and BPH0736 Δ*hsdS* with plasmid pIMAY Δ736*hsdS* isolated from DC10B and strain-specific E. coli Ec_Se736I (expressing BPH0736*hsdMS*). Note that pIMAY Δ736*hsdS* was used because neither pIMAY nor pRAB11 possessed any TRMs. (D) Number of S. epidermidis strain-specific HsdS TRMs present on each plasmid.

To determine the restriction barrier posed by type I RM systems in S. epidermidis and assess the efficiency of PAM as a means of bypassing restriction barriers (Fig. 4), Δ*hsdS* mutants and E. coli hosts for PAM were constructed for S. epidermidis isolates BPH0662, RP62a, and BPH0736. Two different plasmids (pRAB11 [19] and pIMAY [8]) were used in electroporation experiments, as each carried a different number of TRMs recognized by the type I RM systems present in each isolate (Fig. 4D). A clinical ST2 isolate, BPH0662-WT (BPH0662 wild type), was found to have an intractable restriction barrier unless both functional type I RM systems were overcome by complete bypass with PAM in an E. coli host (Ec_Se662I-II) or by deletion of both complete *hsdS* genes (BPH0662 Δ*hsdSI* Δ*hsdSII*) or by a combination of the two approaches (plasmid from Ec_Se662I transferred into BPH0662 Δ*hsdSII* or plasmid from Ec_Se662II transferred into BPH0662 Δ*hsdSI*) (Fig. 4A).

Using our protocol, the type I restriction barrier in RP62a-WT was found to be incomplete. Low numbers of transformants (10^1^ CFU/ml) were obtained with plasmid DNA isolated from DC10B, indicating that bypassing the type IV restriction barrier alone was sufficient to allow genetic manipulation of this strain (Fig. 4B) as previously demonstrated (8). Complete bypass of the single type I RM system in this isolate with E. coli host Ec_SeRP62aI significantly improved electroporation efficiency to 10^4^ CFU/ml, which was equivalent to the complete absence of a functional type I RM system as determined with the RP62a Δ*hsdS* mutant (Fig. 4B). In contrast, when expressing the RP62a *hsdMS* genes from a plasmid in DC10B, Costa et al. were unable to completely bypass the type I RM barrier. This discrepancy was attributed to the presence of additional RM systems with low-frequency methylation (11). However, our results showed that only one type I RM system is present in RP62a, suggesting that the heterologous expression of type I RM systems on a plasmid in DC10B rather than from a single copy of the genes integrated into the chromosome may be suboptimal. Previously, we found that plasmid-based expression of *hsdMS* was unstable and that cells were unable to tolerate the high level of expression required for complete methylation of the target DNA (9).

Clustered regularly interspaced short palindromic repeat (CRISPR) loci confer sequence-directed immunity against phages and other foreign DNA and represent another recognized barrier to horizontal gene transfer in S. epidermidis (20). Our analysis of the CRISPR spacers for RP62a (Table S3D) did not demonstrate the presence of any targets on pSK236 (5.6 kb) as used by Costa et al. or on pRAB11 (6.4 kb) or on pIMAY (5.7 kb) as used in this study that would account for the fact that their electroporation efficiency (10^2^ CFU/ml per 5 μg plasmid DNA) (11) was lower than that determined by our protocol (10^4^ CFU/ml per 5 μg plasmid DNA for both pRAB11 and pIMAY).

Isolate BPH0736-WT was predicted to be naturally restriction deficient due to the interruption of *hsdR* by IS elements (Fig. 1B), but PacBio sequencing demonstrated that it retained functional methylation conferred by an intact *hsdMS* system. Due to the complex and infrequently occurring TRM dictated by the single *hsdS* (Table 3), neither pIMAY nor pRAB11 had any BPH0736-S TRMs present. Therefore, pIMAY bearing the Δ736*hsdS* insertion (pIMAY Δ736*hsdS*) was used as this contained three TRMs (Fig. 4D). BPH0736-WT was functionally confirmed to be restriction deficient, with the same electroporation efficiency (10^4^ CFU/ml) demonstrated for both BPH0736-WT and BPH0736 Δ*hsdS* using plasmid isolated from nonspecific E. coli host DC10B and PAM-tailored mutant Ec_Se736I (Fig. 4C). Further supporting our bioinformatic predictions, like BPH0736, ATCC 12228 (truncated *hsdR* and no *hsdM*; ST8), 1457 (truncated HsdS containing only one TRD, a truncated *hsdR*, and no *hsdM*; ST86), and BPH0710 (truncation at amino acid 81 of HsdS; ST2) were all predicted to have no functional restriction barrier. Similarly to BPH0736, these three strains were transformable at levels on the order of 10^4^ CFU/ml with plasmid isolated from DC10B, suggesting that this was the maximum electroporation efficiency expected for our protocol. A clinical ST2 strain, BPH0676, was also predicted to have no restriction barrier and the complete absence of a type I RM system; however, similarly to BPH0662, the maximum electroporation efficiency achieved was only 10^3^ CFU/ml, suggesting that inherent strain-dependent factors other than type I RM systems, e.g., cell wall thickness (21), impacted the electroporation of these isolates.

Although the data presented above demonstrate that PAM is an efficient method to overcome the type I restriction barrier of S. epidermidis, we observed potential instability with the integration of multiple S. epidermidis *hsdMS* genes of particular TRMs in a DC10B E. coli background. With serial passage of Ec_Se662I-II, the electroporation efficiency of plasmid isolated from this E. coli host into BPH0662 declined from 10^3^ to 10^1^ CFU/ml despite all other experimental parameters remaining the same. This was not observed for any of the E. coli PAM mutants expressing a single *hsdMS* gene, including Ec_Se662I and Ec_Se662II, which maintained high-level methylation (89.65% to 99.90%) of motifs within the genome (12) (Table S3). Illumina sequencing of the Ec_Se662I-II genome confirmed integration of both *hsdMS* genes at the expected chromosomal sites but loss of approximately half of the coding sequence of both *hsdS* genes for the majority of the population sequenced. This instability was hypothesized to be due to the burden of excessive DNA methylation (10,930 sites of heterologous expression of two BPH0662 S. epidermidis type I RM systems in addition to 38,592 sites of endogenous E. coli *dam* methylation) that may interfere with normal cellular function, rendering expression toxic in E. coli. The same likely accounts for the poor electroporation efficiency seen in experiments using PAM for NIH4008 (100-fold lower than that observed for isolates with only a single type I RM system) as reported previously by Costa et al. (11). NIH4008 possesses the same type I RM systems as BPH0662, without the truncation of the orphan *hsdS* gene (Fig. 3). Furthermore, although stable chromosomal integration of three S. aureus *hsdMS* systems in E. coli DC10B (IM93B) was described previously by Monk et al., decreased efficiency of methylation was observed, with only 10,135 of a total of 14,602 TRM sites demonstrating detectable methylation (9).

Collectively, our current and previous (9, 12) data suggest that DC10B E. coli is unlikely to consistently maintain heterologous expression of staphylococcal type I RM systems in the setting of high-frequency methylation (>10,000 sites). This limitation should not impact plasmid electroporation for mutant creation by allelic exchange, which theoretically requires only a single transformant. However, should high-efficiency electroporation be sought (e.g., for direct transposon mutant library selection), then suitable strains can be predicted using genomic data to identify restriction-deficient isolates, such as our newly described reference isolate BPH0736, representing a clinically significant ST2 isolate. Clinical metadata, genome characteristics, CRISPR spacers (when present), *in silico* resistome, an Vitek 2 antibiogram representing clinically relevant antibiotics, and common plasmid selection markers for the seven new reference isolates and BPH0662 are shown in Table S3. Metadata and sequencing accession numbers for mutant isolates are listed in Table S5.

10.1128/mBio.02451-19.9 TABLE S5 S. epidermidis Δ*hsdS* and E. coli plasmid artificial modification mutant sequencing accession data. (A) PacBio sequencing. (B) PacBio methylation. (C) Illumina sequencing. Download TABLE S5, XLSX file, 0.02 MB. Copyright © 2019 Lee et al. 2019 Lee et al. This content is distributed under the terms of the Creative Commons Attribution 4.0 International license.

### Phage transduction of plasmid is subject to type I restriction.

**FIG 5:**
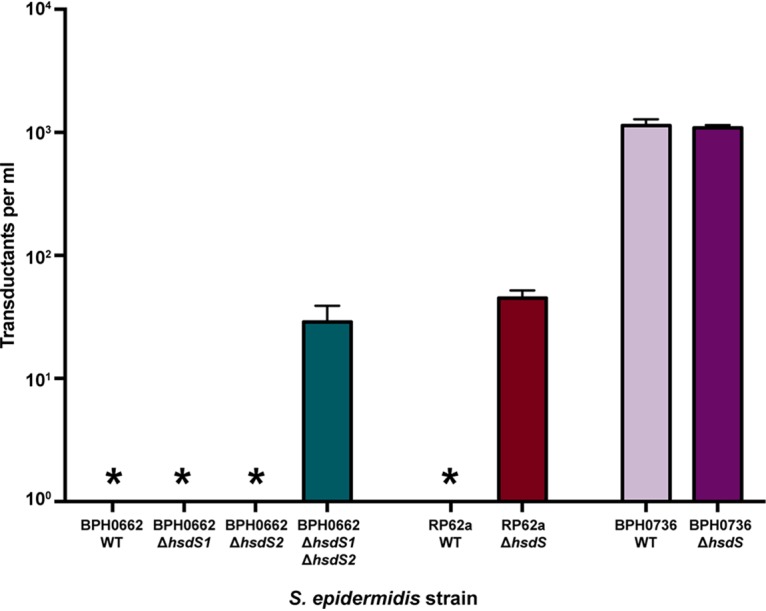
S. epidermidis phage transduction is subject to type I restriction. Biological triplicate data were determined for phage transduction of Φ187-pRAB11 lysate transduced into S. epidermidis wild-type (WT) and Δ*hsdS* mutant strains. Error bars represent means ± standard deviations of results from three independent experiments. *, no transductants.

Phage transduction is an alternative method for the genetic manipulation of S. epidermidis. In particular, S. aureus ST395 lineage-specific Φ187 shares wall teichoic acid (WTA) receptors with S. epidermidis (22, 23). Depending on the incidental packaging of plasmid introduced into a restriction-deficient intermediary host, S. aureus PS187 Δ*hsdR* Δ*sauPSI*, with Φ187 phage machinery (24), the method can be used to transduce a number of CoNS strains but is not universally applicable to all S. epidermidis isolates (23). The observed ability of ST395 S. aureus to exchange DNA with some CoNS strains led Winstel et al. to conclude that overlap of the DNA methylation of ST395 S. aureus and that of CoNS strains that share the same WTA receptors may exist (22, 23). Results of phage Φ187 transduction experiments performed using our WT isolates and Δ*hsdS* mutants for BPH0662, RP62a, and BPH0736 representing the transfer of pRAB11 are shown in Fig. 5. These experiments demonstrated that even if successfully transduced into a S. epidermidis isolate, plasmids are still subject to degradation by type I RM systems if they bear a recognized TRM. However, in BPH0736 (absent type I restriction) or in mutant strains in which systems have been rendered inactive, transduced plasmid remains viable. The methylome for ST395 S. aureus has not been characterized; however, the draft genome sequence for PS187 (GCA_000452885.1) indicates that the two type I RM systems in this isolate are identical to those in S. aureus isolate JS395 (ST1093, belonging to CC395 [25]). We predicted the methylome of the isolate to include G**A**GN_6_TCG (same as AUS0325-MS2) and another unknown TRM (Fig. 2) (Table 2). The results of our experiments and analyses of the diversity of S. epidermidis type I RM systems suggest that successful phage transduction of some S. epidermidis isolates with Φ187 is more likely related to the absence of a functional system than to the presence of a methylome shared with ST395 S. aureus. This is further supported by data from experiments performed by Winstel et al. (23) in which Φ187 was found to be able to transduce pTX15 (26) only into RP62a and not into pKOR1 (27). On the basis of our characterized RP62a TRM, we determined that pTX15 possesses no RP62a motifs whereas pRAB11 and pKOR1 each bear four motifs, explaining why neither plasmid is transducible into RP62a-WT.

A temperature-sensitive plasmid, pIMAY, is frequently used for allelic exchange in staphylococci due to the presence of inducible *secY* antisense counterselection and the lower likelihood of unintended mutations (that occurs with pKOR1) as integrants are selected at 37°C instead of 43°C (28). However, we found that Φ187 was not capable of transducing pIMAY into any of the tested strains, including the Δ*hsdS* mutants and naturally restriction-deficient BPH0736. We hypothesized this was due to the low copy number of pIMAY in staphylococci, resulting in low levels of incidental packaging of the plasmid within Φ187, compared to high-copy-number plasmid pRAB11. Other limitations of Φ187 transduction include a recommendation to use plasmids of <10 kb (24); however, that should not have impacted pIMAY (5.7 kb), which is smaller than pRAB11 (6.4 kb). Although a simplified harvesting and infection protocol was used compared to that described by Winstel et al. (24), we achieved an efficiency of 10^4^ transductants per ml with pRAB11, equivalent to their anticipated results of 10^1^ to 10^4^ (24), in restriction-deficient S. epidermidis strain BPH0736. Of note, the levels of efficiency of both Δ*hsdS* mutants, BPH0662 Δ*hsdSI* Δ*hsdSII* and RP62a Δ*hsdS*, were 2 logs lower than those measured for BPH0736 (Fig. 5), further supporting the theory that strain-dependent factors beyond the barriers posed by type I RM systems and WTA are present in these backgrounds.

### Conclusions.

Our results demonstrate marked differences between the type I RM systems in S. aureus and S. epidermidis, which had hitherto been assumed to share the same characteristics (11). These differences are predominantly attributable to the arrangement and genome location of the S. epidermidis type I system as a complete three-gene operon, which we hypothesize to be mobilized by *ccr*. Localization of the operon in a highly plastic region of the chromosome increases the likelihood of horizontal transfer of these complete systems between S. epidermidis strains as well as to other staphylococci. This results in a lack of lineage specificity and a higher probability of spontaneous interruption of component genes. This is in contrast to S. aureus, where the type I systems are typically arranged as one *hsdR* and two *hsdMS* genes located apart from one another in stable regions of the chromosome. The evolutionary impact of these differences in the type I RM systems of S. epidermidis and S. aureus are unknown and warrant future research. The diversity of S. epidermidis type I RM systems that do not strictly adhere to ST/CC groupings indicates that genetic manipulation of S. epidermidis requires tailoring for each isolate of interest. Attempting electroporation without genomic analysis of the methylome could be successful, as our analyses found that 38% of S. epidermidis strains did not possess a type I RM system, and not all systems pose an intractable barrier (e.g., RP62a). However, some isolates such as internationally disseminated, almost pan-drug-resistant clone BPH0662 have complex and absolute type I restriction barriers.

We have demonstrated that PAM using a DC10B E. coli host is a simple and effective means to bypass the type I RM barrier in S. epidermidis, with plasmid transfer efficiency equivalent to that seen in the complete absence of type I RM systems. The decreasing cost and ready availability of whole-genome sequencing has made the sequencing of isolates planned for mutagenesis and their mutant derivatives commonplace and a practice that is recommended to ensure the absence of acquired secondary mutations (29). If the genome sequence of an isolate is known, then its methylome and ability to be transformed can be predicted as follows. (i) Does the isolate possess an intact type I RM system? If not, type I methylation would not be expressed and the isolate should be inherently transformable. (ii) Each complete type I RM system within a genome should be functional. For an HsdS protein with known TRMs, the presence of the TRMs on a vector would likely prevent electroporation. (iii) Orphaned, complete *hsdS* genes may be expressed in the absence of an adjacent *hsdM* if the associated *hsdM* allele is present elsewhere in the genome. In view of the guidelines presented above, when designing an E. coli PAM host, to ensure complete recapitulation of the endogenous type I methylome, we recommend including all complete *hsdMS* genes and any complete orphan *hsdS* genes from the S. epidermidis strain to be manipulated.

The 247 genomes that we analyzed are by no means an exhaustive representation of all S. epidermidis, and additional examples of type I RM systems will undoubtedly be catalogued as further sequencing of this organism is performed. However, this genomic sampling and our functional data were sufficient to draw the conclusions presented above. In view of the identified complexities associated with the genetic manipulation of S. epidermidis, the BPH0736 reference isolate should prove particularly useful. A clinical ST2 isolate that is representative of international circulating clones (5), BPH0736 is naturally type I restriction deficient due to the spontaneous interruption of *hsdR*, rendering it highly amenable to both electroporation and phage transduction and making it an ideal strain for future molecular studies.

## MATERIALS AND METHODS

### Media and reagents.

Bacterial strains, plasmids, and oligonucleotides used in this study are listed in Table S6 in the supplemental material. S. epidermidis were routinely cultured at 37°C in brain heart infusion (BHI) broth (Difco). See Text S1 in the supplemental material for detailed descriptions of culture media, antibiotics, and enzymes.

10.1128/mBio.02451-19.1 TEXT S1 Supplementary Methods. Download Text S1, DOCX file, 0.04 MB. Copyright © 2019 Lee et al. 2019 Lee et al. This content is distributed under the terms of the Creative Commons Attribution 4.0 International license.

10.1128/mBio.02451-19.10 TABLE S6 Strains, plasmids, and oligonucleotides used in this study. Download TABLE S6, DOCX file, 0.04 MB. Copyright © 2019 Lee et al. 2019 Lee et al. This content is distributed under the terms of the Creative Commons Attribution 4.0 International license.

### Genome sequencing and analysis.

The genome sequencing and analysis procedures are described in Text S1.

### Electroporation.

Early (8-h)-stationary-phase cultures of S. epidermidis grown in 10 ml of B media (BM) were added to 90 ml of fresh, prewarmed BM. Cultures were reincubated to an optical density at 600 nm (OD_600_) of between 0.8 and 0.9 and chilled in an ice slurry for 10 min. Cells were harvested at 3,900 × *g* for 5 min at 4°C in a swinging bucket rotor, and the cell pellet was resuspended in 100 ml of autoclaved, ice-cold water. Centrifugation was repeated, and the pellet was resuspended in 50 ml of autoclaved ice-cold water. Cells were centrifuged and successively resuspended in 20 ml, 10 ml, and 250 μl of autoclaved ice-cold 10% (wt/vol) glycerol. Equal aliquots (50 μl) were frozen at –80°C. Prior to electroporation, cells were thawed on ice for 5 min and then at room temperature for 5 min. Following centrifugation at 5,000 × *g* for 1 min, cells were resuspended in 50 μl of 10% glycerol–500 mM sucrose (filter sterilized). Pellet paint (Novagen) precipitated plasmid DNA was added to the cells, and then the cells were transferred into a 1-mm-path length electroporation cuvette (Bio-Rad) and pulsed at 21 kV/cm, 100 Ω, and 25 μF at room temperature. Routinely, 5 μg of plasmid DNA was used, with concentrations determined by fluorometric assay (Qubit 2.0; Life Technologies). Cells were incubated in 1 ml of BHI broth supplemented with 500 mM sucrose (filter sterilized) at 28°C for 2 h prior to plating on BHI agar (BHIA) containing chloramphenicol (10 μg/ml).

### Construction of Ec_Se736I and Ec_SeRP62aI E. coli hosts.

E. coli mutants expressing the relevant S. epidermidis type I RM systems in a DC10B background were created as previously described (8, 9, 12) using the primers listed in Table S6. Details of the methodology are provided in Text S1.

### Construction of S. epidermidis Δ*hsdS* mutants.

The pIMAY(Δ*hsdS*) vectors were constructed using amplification by overlap extension PCR (30) with the A/B/C/D primer sets specified for each strain in Table S6, cloning into the pIMAY vector backbone, and subsequent cloning of the insertion into the vector. Mutant selection and screening were conducted as previously described (5). Details of the methodology are provided in Text S1.

### Harvesting Φ187 plus pRAB11/pIMAY lysate from S. aureus PS187 Δ*hsdR* Δ*sauPSI*.

Φ187 containing pRAB11/pIMAY was harvested from S. aureus PS187 Δ*hsdR* Δ*sauPSI* using a protocol adapted from Winstel (24). See Text S1 for detailed methodology.

### Φ187 plus pRAB11/pIMAY transduction of S. epidermidis.

A phage transduction protocol was adapted a method described previously by Foster et al. (31). Details of the methodology are provided in Text S1.

### Data accessibility.

Isolate BPH0662 has been deposited with the NCTC (NCTC accession no. 14219). The data sets supporting the results of this article are available from NCBI (BioProject PRJNA532483) and ENA (BioProject PRJEB35032) (sequencing and closed genome assemblies) and Figshare (https://melbourne.figshare.com/articles/Sa_HsdS_48_fasta/7986956 [S. aureus HsdS]; https://melbourne.figshare.com/articles/Se_HsdS_31_fasta/7986911 [S. epidermidis HsdS]; https://melbourne.figshare.com/articles/Se_HsdM_6_fasta/7986827 [S. epidermidis HsdM]; https://melbourne.figshare.com/articles/Se_HsdR_5_fasta/7986893 [S. epidermidis HsdR]).
