# Supplementary material for: Mining the Methylome Reveals Extensive Diversity in Staphylococcus epidermidis Restriction Modification
Source: mBio. 2019 Dec 17;10(6):e02451-19. doi: 10.1128/mBio.02451-19 (PMC6918075; doi:10.1128/mBio.02451-19)
Supplement: FIG S1 [file mBio.02451-19-sf001.pdf]

A. *Staphylococcus epidermidis* complete genomes type I RM systems

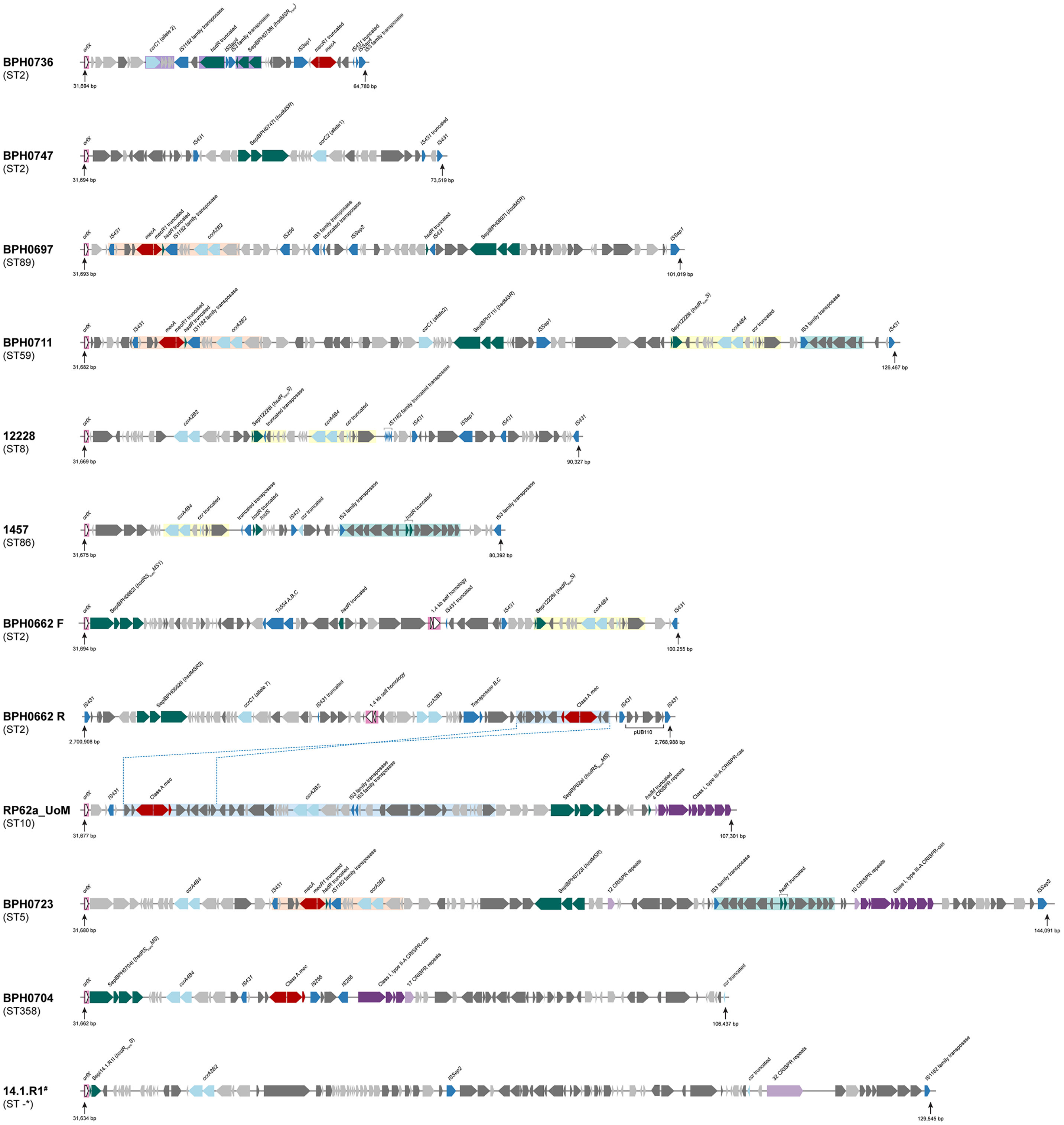

B. *Staphylococcus aureus* imported type I RM systems

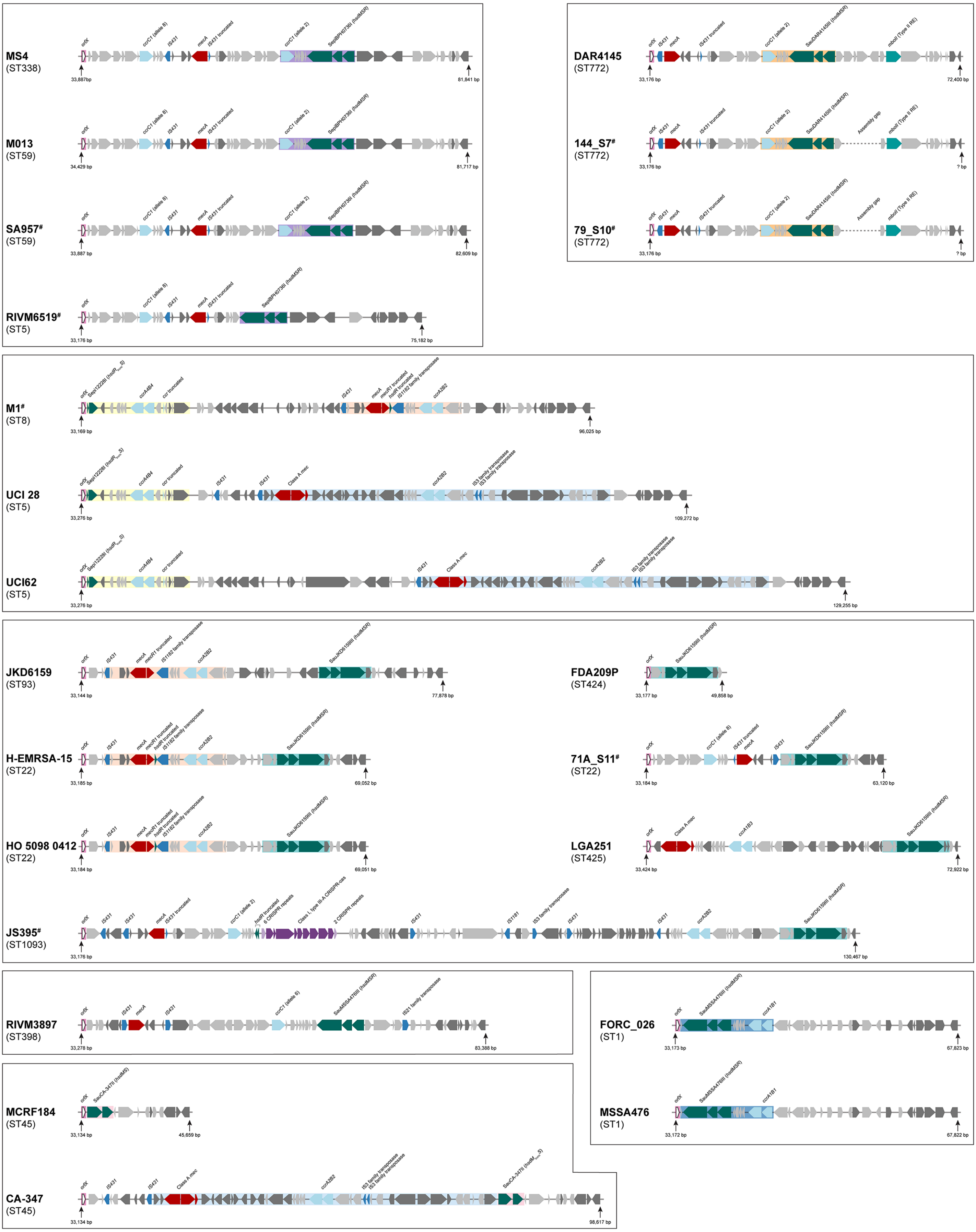

Figure S1. A hypothesised role for cassette chromosome recombinase (*ccr*) in the mobilisation of *S. epidermidis* and *S. aureus* type I restriction modification systems. A. Complete *S. epidermidis* genomes with type I RM systems. **B.** *S. aureus* genomes with imported type I RM systems. Genomes are orientated forwards starting at *dnaA*. #NCBI uploaded genome does not start at *dnaA*. *Strain not classifiable by existing MLST scheme.
