# Supplementary material for: Mining the Methylome Reveals Extensive Diversity in Staphylococcus epidermidis Restriction Modification
Source: mBio. 2019 Dec 17;10(6):e02451-19. doi: 10.1128/mBio.02451-19 (PMC6918075; doi:10.1128/mBio.02451-19)
Supplement: FIG S2 [file mBio.02451-19-sf002.pdf]

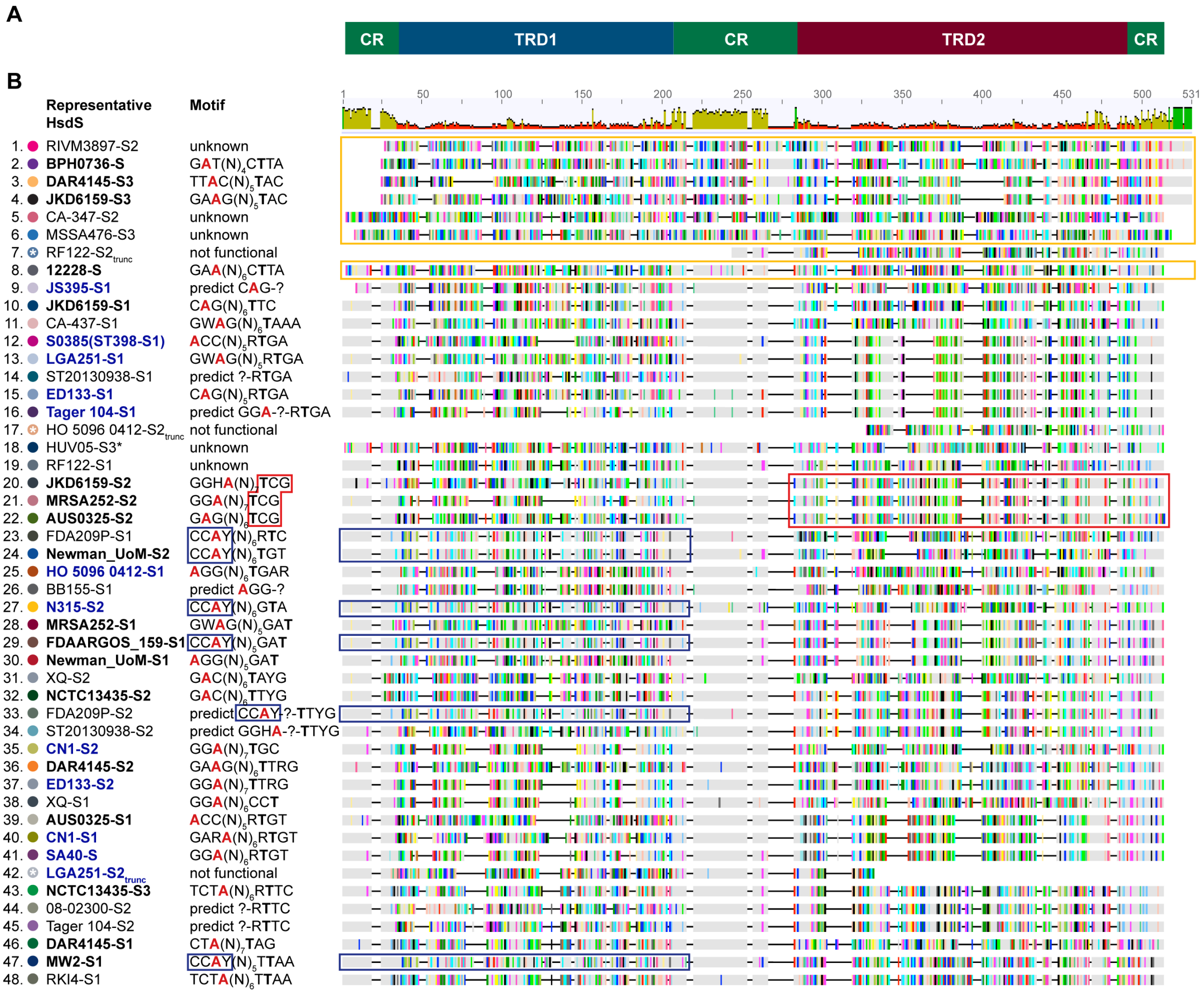

A
CR
TRD1
CR
TRD2
CR
B
Representative HsdS
Motif
1
50
100
150
200
250
300
350
400
450
500
531
1. RIVM3897-S2 unknown
2. BPH0736-S GAT(N)$_4$CTTA
3. DAR4145-S3 TTAC(N)$_5$TAC
4. JKD6159-S3 GAAG(N)$_5$TAC
5. CA-347-S2 unknown
6. MSSA476-S3 unknown
7. RF122-S2$_{trunc}$ not functional
8. 12228-S GAA(N)$_6$CTTA
9. JS395-S1 predict CAG-?
10. JKD6159-S1 CAG(N)$_6$TTC
11. CA-437-S1 GWAG(N)$_6$TAAA
12. S0385(ST398-S1) ACC(N)$_5$RTGA
13. LGA251-S1 GWAG(N)$_5$RTGA
14. ST20130938-S1 predict ?-RTGA
15. ED133-S1 CAG(N)$_5$RTGA
16. Tager 104-S1 predict GGA-?-RTGA
17. HO 5096 0412-S2$_{trunc}$ not functional
18. HUV05-S3* unknown
19. RF122-S1 unknown
20. JKD6159-S2 GGHA(N)$_7$TCG
21. MRSA252-S2 GGA(N)$_7$TCG
22. AUS0325-S2 GAG(N)$_6$TCG
23. FDA209P-S1 CCAY(N)$_6$RTC
24. Newman_UoM-S2 CCAY(N)$_6$TGT
25. HO 5096 0412-S1 AGG(N)$_6$TGAR
26. BB155-S1 predict AGG-?
27. N315-S2 CCAY(N)$_6$GTA
28. MRSA252-S1 GWAG(N)$_5$GAT
29. FDAARGOS_159-S1 CCAY(N)$_5$GAT
30. Newman_UoM-S1 AGG(N)$_5$GAT
31. XQ-S2 GAC(N)$_6$TAYG
32. NCTC13435-S2 GAC(N)$_6$TTYG
33. FDA209P-S2 predict CCAY-?-TTYG
34. ST20130938-S2 predict GGHA-?-TTYG
35. CN1-S2 GGA(N)$_7$TGC
36. DAR4145-S2 GAAG(N)$_6$TTRG
37. ED133-S2 GGA(N)$_7$TTRG
38. XQ-S1 GGA(N)$_6$CCT
39. AUS0325-S1 ACC(N)$_5$RTGT
40. CN1-S1 GARA(N)$_6$RTGT
41. SA40-S GGA(N)$_6$RTGT
42. LGA251-S2$_{trunc}$ not functional
43. NCTC13435-S3 TCTA(N)$_6$RTTC
44. 08-02300-S2 predict ?-RTTC
45. Tager 104-S2 predict ?-RTTC
46. DAR4145-S1 CTA(N)$_7$TAG
47. MW2-S1 CCAY(N)$_5$TTAA
48. RKI4-S1 TCTA(N)$_6$TTAA

Figure S2. Alignment of *S. aureus* HsdS variants. A. Structure of an HsdS allele with conserved regions (CRs) flanking two variable regions known as target recognition domains (TRD1 & TRD2). **B.** Each TRD typically specifies three to four defined base pairs including a methylated adenine residue (red **A**; **T** = complementary partner to methylated adenine residue); with a four to seven base pair non-specific spacer (N) between the two defined halves, collectively these TRDs determine the full target recognition motif (TRM) specified by an HsdS variant. HsdS names in bold black font have motifs determined by PacBio sequencing of the isolate after which the representative HsdS was named. HsdS names in bold blue font have motifs determined by DNA cleavage with purified restriction enzyme. Alignments of the identified variants of *S. aureus* HsdS are shown adjacent to their TRMs, each formed by a different TRD pairing. Scale above alignments indicates the position in the consensus alignment with mean pairwise identity at each site graphed (green = 100% identity; khaki = 30-100%; red <30%). Blue (TRD1) and red (TRD2) outlines highlight examples of TRDs that recur within the alignments and the TRM base pairs they define. Yellow boxes highlight alignments of HsdS imported into *S. aureus* on Staphylococcal cassette chromosome elements.
