# Supplementary material for: Mining the Methylome Reveals Extensive Diversity in Staphylococcus epidermidis Restriction Modification
Source: mBio. 2019 Dec 17;10(6):e02451-19. doi: 10.1128/mBio.02451-19 (PMC6918075; doi:10.1128/mBio.02451-19)
Supplement: FIG S3 [file mBio.02451-19-sf003.pdf]

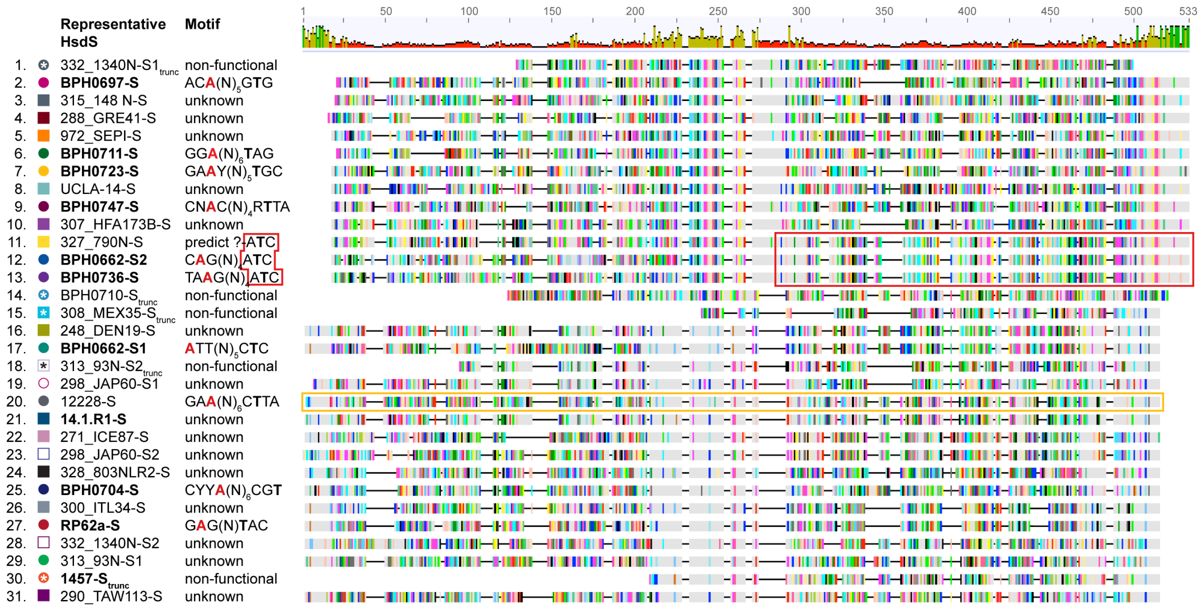

Figure S3. Alignment of *S. epidermidis* HsdS variants. HsdS names in bold black font have motifs determined by PacBio sequencing of the isolate after which the representative HsdS was named. Target recognition motifs (TRMs) (when known) and amino acid alignments are shown adjacent. Scale above alignments indicates the position in the consensus alignment with mean pairwise identity at each site graphed (green = 100% identity; khaki = 30-100%; red <30%). Red outline highlights an example of a target recognition domains that recurs within the alignments and the TRM base pairs they define. Yellow box highlights the 12228-S alignment, this HsdS variant was found to be present in both *S. aureus* and *S. epidermidis* and shared conserved regions with the *S. aureus* HsdS variants located within stable chromosomal islands (Figure S2).
