# Supplementary material for: Mining the Methylome Reveals Extensive Diversity in Staphylococcus epidermidis Restriction Modification
Source: mBio. 2019 Dec 17;10(6):e02451-19. doi: 10.1128/mBio.02451-19 (PMC6918075; doi:10.1128/mBio.02451-19)
Supplement: TABLE S4 [file mBio.02451-19-st004.docx]

**Determination of the HsdM variant that interacts with 12228 HsdS.**

| 1. Three type I systems were identified in NIH4008 (1) without assignment of the TRM to their associated *hsdS*, for which only draft genome sequencing was available (2). |
| --- |
| 1. Expression of 12228-S by this isolate indicated that at least one of the two HsdM present were capable of interacting with 12228-S. |
| 1. The same three type I RM systems are present in closed reference genome BPH0662 (3), for which the TRM of the two complete type I RM systems were assigned through heterologous expression in an *E. coli* host (3). |
| 1. The 12228-S is non-functional in BPH0662 due to a SNP (64∆A) resulting in truncation after the first 21 amino acids. |
| 1. Our analysis of the BPH0711 methylome indicated expression of only one TRM despite 12228-S also being present, since BPH0711-M has 98.0% pairwise identity with BPH0622-M2, and was not able to interact with 12228-S, BPH0662-M1 was determined to interact with 12228-S. |

**References**

1. Costa SK, Donegan NP, Corvaglia A-R, Francois P, Cheung AL. 2017. Bypassing the restriction system to improve transformation of *Staphylococcus epidermidis*. J Bacteriol 199:e00271–17.

2. Conlan S, Mijares LA, NISC Comparative Sequencing Program, Becker J, Blakesley RW, Bouffard GG, Brooks S, Coleman H, Gupta J, Gurson N, Park M, Schmidt B, Thomas PJ, Otto M, Kong HH, Murray PR, Segre JA. 2012. *Staphylococcus epidermidis* pan-genome sequence analysis reveals diversity of skin commensal and hospital infection-associated isolates. Genome Biol 13:R64.

3. Lee JYH, Monk IR, Pidot SJ, Singh S, Chua KYL, Seemann T, Stinear TP, Howden BP. 2016. Functional analysis of the first complete genome sequence of a multidrug resistant sequence type 2 *Staphylococcus epidermidis*. Microb Genom 2:e00007.
