# Supplementary material for: Mining the Methylome Reveals Extensive Diversity in Staphylococcus epidermidis Restriction Modification
Source: mBio. 2019 Dec 17;10(6):e02451-19. doi: 10.1128/mBio.02451-19 (PMC6918075; doi:10.1128/mBio.02451-19)
Supplement: TEXT S1 [file mBio.02451-19-s0001.docx]

**Media and reagents.** Bacterial strains, plasmids and oligonucleotides used in this study are listed in Table S6. *S. epidermidis* were routinely cultured at 37^o^C in brain heart infusion broth (BHI)(Difco). For electroporation, *S. epidermidis* were grown in B media (BM) (1% Bacto^TM^ Proteose Peptone No.3, 0.5% yeast extract, 0.5% NaCl, 0.1% K_2_HPO_4_, 0.1% glucose). *E. coli* was routinely cultured in L broth (LB) (1% tryptone, 0.5% yeast extract, 0.5% NaCl). Staphylococci infected with Φ187 were routinely cultured in LB. For growth on agar, BHI or LB were solidified with 1.5% agar, to yield BHIA and LBA, respectively. The following antibiotics were purchased from Sigma Aldrich and used at the specified concentrations: chloramphenicol (Cm) 10 μg/ml in *S. epidermidis*, 15 μg/ml in *E. coli*; ampicillin (Amp) 100 μg/ml in *E. coli*; kanamycin (Kan) 50 μg/ml in *E. coli.* Oligonucleotides were purchased from Integrated DNA Technologies. Isolation of genomic DNA, plasmid purification, gel extraction and colony PCR were performed as previously described (1). Restriction enzymes and Phusion DNA polymerase were purchased from New England Biolabs. Phire Hotstart DNA polymerase was purchased from Thermofisher.

**Genome sequencing.** Single molecule real-time (SMRT) sequencing was performed on a PacBio RS II instrument with subsequent analysis performed using SMRT Analysis v2.3.0.140936. The RS_HGAP_Assembly.3 algorithm was used for *de novo* assembly with trimmed Illumina reads used to correct homopolymer errors using Snippy v3.2 (2). Illumina reads were assembled using SPAdes v3.7.1 (3) and the resulting contigs were screened for small plasmids that would be lost during PacBio size selection. Final assemblies for each new reference genome were independently confirmed using Unicycler v0.3.0 (4). For methylome analysis, each newly closed genome was used as its own reference with the RS_Modification_and Motif_Analysis.1 algorithm. Draft genomes were sequenced using on an Illumina Nexseq platform with Nextera XT libraries, as per manufacturer’s instructions.

**Genome analysis.** All publicly available, *S. epidermidis* genome raw reads in NCBI SRA were downloaded (4^th^ November 2016). Exclusion criteria were as follows: sequencing performed on a Roche 454 or Ion Torrent platform (per Genbank metadata); single end reads; sequencing depth <30x; mutagenesis isolate pairs; organism not *S. epidermidis* or sequencing of mixed bacterial species as determined by Kraken v0.10.15beta (5); >300 assembled contigs; raw reads not associated with publications and lacking metadata. When isolates were identified as exact genetic replicates, a single representative was used in analyses. Trimming, assembly, genome annotation and *in silico* MLST determination was performed as previously described (1). Isolate VCU036 with a previously unspecified ST (6), was independently reanalysed using a second method, MLST v1.8(7). CRISPR type and direct repeat and spacer sequences were determined using CRISPRCasFinder (8). Maximum-likelihood core-SNP phylogeny of *S. epidermidis* isolates and hierarchical Bayesian Analysis of Population Structure cluster modelling of the resultant tree was determined as previously described (1), using the closed BPH0736 genome as a reference. All publicly available, closed *S. aureus* genomes available were downloaded (2^nd^ February 2017). Mutant derivatives and repeat sequencing of serial isolates over the course of an infection were excluded from analyses. Sa_Newman_UoM (GCA_900092595.1) (9) was used as the reference genome to generate a maximum likelihood core-SNP phylogeny as described (1).

To determine the diversity of HsdS in *S. epidermidis* and *S. aureus*, each of the ten *S. epidermidis* (Table 3) and 32 *S. aureus* (Table 2) HsdS translations with characterised TRMs were used as a reference for individual tblastn queries against the collected assemblies using BLAST v2.6.0 (10) with an e-value cutoff of 1e^-6^, and low complexity filtering disabled (-F F); the output of which was aligned with Clustal Omega v1.2.4 (11). To validate this approach, all *S. epidermidis* and *S. aureus* genomes were manually screened for the presence of type I RM systems or component genes using Geneious v7.1.8 (12) and type I systems were manually extracted as contiguous operons and full individual genes. The complete HsdS translations from all isolates were aligned with Clustal Omega, a shared amino acid identity of <90% was used to classify separate variants of HsdS. The diversity of HsdR and HsdM subunits in *S. aureus* and *S. epidermidis* were determined using this same method. In the case of closed genomes, systems were numbered relative to location after *dnaA*.

**Electroporation.** Early stationary phase cultures (8 h) of *S. epidermidis* grown in 10 ml of BM were added to 90 ml of fresh, prewarmed BM. Cultures were reincubated to an OD_600_ between 0.8 - 0.9 and chilled in an ice slurry for 10 min. Cells were harvested at 3,900*xg* for 5 min at 4ºC in a swinging bucket rotor and the cell pellet resuspended in 100 ml of autoclaved, ice-cold water. Centrifugation was repeated, and the pellet resuspended in 50 ml of autoclaved ice-cold water. Cells were centrifuged and successively resuspended in 20 ml, 10 ml then 250 μl of autoclaved ice-cold 10% (weight/volume) glycerol. Equal aliquots (50 μl) were frozen at -80^o^C. Prior to electroporation, cells were thawed on ice for 5 min, then at room temperature for 5 min. Following centrifugation at 5,000 *xg* for 1 min, cells were resuspended in 50 μl of 10% glycerol with 500 mM sucrose (filter sterilised). Pellet paint (Novagen) precipitated plasmid DNA was added to the cells, and transferred into a 1 mm electroporation cuvette (Bio-Rad) and pulsed at 21 kV/cm, 100 Ω, 25 μF at room temperature. Routinely, 5 μg of plasmid DNA was used, with concentration determined by fluorometric assay (Qubit 2.0; Life Technologies). Cells were incubated in 1 ml of BHI supplemented with 500 mM sucrose (filter sterilised) at 28^o^C for 2 h prior to plating on BHIA containing Cm 10 μg/ml.

**Construction of Ec_Se736I and Ec_SeRP62aI *E. coli* hosts.** *E. coli* mutants expressing the relevant *S. epidermidis* type I RM systems in a DC10B background were created using methods previously described (13, 14). The *hsdMS* system from BPH0736 was amplified with primers 736MS-F (incorporating Pxyl/tetO promoter)/IM201. Plasmid pKD4 (15) was used as a template for the amplification of the Kan resistance marker flanked by flippase recognition target (FRT) sites with primers IM202/IM203 (product pKD4). The *hsdMS* and pKD4 products were gel extracted and joined by spliced overlap extension (SOE) PCR using primers IM199/203 (primer tails contained 50 bp homology for integration into the intergenic region between *essQ* and *cspB* in the DC10B chromosome). The *hsdMS*-pKD4 amplicon was pellet paint (Novagen) precipitated and electroporated into DC10B containing pKD46 (15), made competent as previously described (16). Transformants were selected for on L agar Cm10. Colony PCR with primers IM179/IM180 was used to screen for recombination at the integration site, positive clones were grown overnight at 43^o^C to promote loss of pKD46. To excise the pKD4 product, strains were transformed with pCP20 (17) at 28^o^C, single colony purified at 28^o^C, then grown overnight at 43^o^C to promote plasmid loss. To confirm excision of the Kan resistance marker and the loss of pCP20, cells were patch plated on L agar Kan and L agar Cm, respectively. The resulting Kan^S^, Cm^S^ strain containing BPH0736 *hsdMS* (with Pxyl/tetO promoter promoter) was named Ec_Se736I.

The *E. coli* Ec_SeRP62aI mutant was constructed from *E. coli* DC10B using the following primer sets: IM365/266 for the amplification of RP62a *hsdMS* from *S. epidermidis* RP62a, which were then ligated into complementary digested (NcoI/PstI) plasmid pIMK2 (18); Primers IM367/IM266 were used to amplify *hsdMS* with Phelp promoter from pIMK2(*hsdMS*) with subsequent steps following the same protocol described above. Primers IM360/IM366 were used to for amplify the FRT-*kan*-FRT from pKD4 (product pKD4); IM366/IM367 (containing tails with 50 bp homology for integration into the intergenic region between *gidB* and *atpI* in the DC10B chromosome) for SOE PCR of *hsdMS*-pKD4. Primers IM434/IM435 were used to confirm recombination at integration site.

**Construction of *S. epidermidis* ∆*hsdS* mutants.** The pIMAY(Δ*hsdS*) vectors were constructed as follows: *∆hsdS* inserts (designed to delete the complete *hsdS* gene) were constructed using PCR-driven overlap extension (19) and the A/B/C/D primer sets specified for each strain in Table S6; cloning of the pIMAY vector backbone and subsequent cloning of the insert into the vector was as previously described (1). Transformation of pIMAY(Δ*hsdS*) into the relevant DC10B derived *E. coli* host for PAM, plus the subsequent selection of transformants was as described (1). Integration and excision of pIMAY(*∆hsdS*) was performed as described (16), with the exception of concurrent (rather than sequential) colony PCRs: for loss of replicating plasmid (with MCS primers IM3/IM4); and chromosomal integration of the plasmid upstream (relevant Δ*hsd* out forward/IM3) and downstream (IM4/ relevant Δ*hsd* out reverse). Post 1 μg/ml anhydrotetracycline (ATc, Sigma) counter selection, large colonies were patch-plated on BHIA plus ATc1, and BHIA containing Cm10, with successful mutants bearing a Cm^S^ phenotype and loss of the type I restriction barrier. Draft genomes of successfully created mutant strains in *E. coli / S. epidermidis* were sequenced and assembled as above to confirm the insertion/deletion of the *hsdMS/hsdS* at the expected site and determine if any unintended mutations were present. Variant calling between the mutants to their respective parental strains was performed using Snippy v3.2 (2). Mutants and their corresponding parental strains were subjected to phenotypic testing to determine the barrier to genetic manipulation presented by each individual type I RM system.

**Construction of *S. epidermidis* ∆*hsdS* mutants.** The pIMAY(Δ*hsdS*) vectors were constructed as follows: *∆hsdS* inserts (designed to delete the complete *hsdS* gene) were amplified by overlap extension PCR (19) with the A/B/C/D primer sets specified for each strain in Table S6; cloning into the pIMAY vector backbone and subsequent cloning of the insert into the vector was conducted as previously described (1). Transformation of pIMAY(Δ*hsdS*) into the relevant *E. coli* host for PAM and the subsequent selection of transformants was as previously described (1). Integration and excision of pIMAY(*∆hsdS*) was performed as previously described (16), with the exception of concurrent (rather than sequential) colony PCRs: for loss of replicating plasmid (with MCS primers IM3/IM4); and chromosomal integration of the plasmid upstream (relevant Δ*hsd* out forward/IM3) and downstream (IM4/ relevant Δ*hsd* out reverse). Post 1 μg/ml anhydrotetracycline (ATc1, Sigma) counter selection, large colonies were patch-plated on BHIA plus ATc1, and BHIA containing Cm10, with successful mutants bearing a Cm^S^ phenotype and loss of the type I restriction barrier. Draft genomes of successfully created mutant strains in *E. coli/S. epidermidis* were sequenced and assembled as above to confirm the insertion/deletion of the *hsdMS/hsdS* at the expected site and determine if any unintended mutations were present. Variant calling between the mutants to their respective parental strains was performed using Snippy v3.2 (2). Mutants and their corresponding parental strains were subject to phenotypic testing to determine the barrier to genetic manipulation presented by each individual type I RM system.

**Harvesting Φ187 + pRAB11/pIMAY lysate from *S. aureus* PS187Δ*hsdR*Δ*sauPSI***

To harvest Φ187 containing pRAB11/pIMAY, an overnight culture of *S. aureus* PS187Δ*hsdR*Δ*sauPSI* (20) containing pRAB11 was diluted to an OD_600_ 1:100 in 50 ml of prewarmed LB plus 10 mM CaCl_2_/Cm10 and grown at 37^o^C for pRAB11 (28^o^C for pIMAY) with aeration at 200 rpm for 4 h (final OD_600_ 2.5). In 15 ml tubes, 300 μl aliquots of the 4 h *S. aureus* PS187Δ*hsdR*Δ*sauPSI* containing pRAB11/pIMAY culture were infected with 200 μl of 10^-1^ to 10^-6^ serial dilutions of Φ187 lysate. Tubes were incubated for 45 min at room temperature, then 10 ml of molten top agar LBA plus 10 mM CaCl_2_/Cm10 (0.35% agar) was added to the infection reaction and the mixture poured over LBA plus 10 mM CaCl_2_/Cm10 base agar (1.5% agar). Plates were incubated at 37^o^C overnight without inversion. The top agar layer of plates with cotton wool appearance (10^-4^ to 10^-5^ dilution) were transferred into 15 ml tubes with a sterile spreader and centrifuged at 15,000 *xg* for 10 min at 4^o^C. Supernatant was filter sterilised through a 0.22 μM filter and stored at 4^o^C. Phage titer was determined by plaque assay as previously described(20), using LBA rather than BM agar.

**Φ187 + pRAB11/pIMAY transduction of *S. epidermidis.*** A phage transduction protocol was adapted from Foster (21). A 25 ml overnight culture of the target *S. epidermidis* strain was harvested at 7,000 *xg* for 10 min, then resuspended in 1 ml L broth supplemented with 10 mM CaCl_2_ (LBC). In 15 ml tubes, 500 μl of suspension was added to: 1.0 ml of LBC and 500 μl Φ187-plasmid lysate (10^11^ plaque forming units); and control of 500 μl of suspension plus 1.5 ml of LBC. Tubes were incubated in a 37^o^C water bath for 25 min, then at 37^o^C a further 15 min with aeration at 200 rpm. To each tube, 1 ml of ice cold 0.02 M sodium citrate was added, and the cells were harvested at 7,000 *xg* for 10 min at 4^o^C. The cell pellet was resuspended in 1 ml of ice cold 0.02 M sodium citrate and incubated on ice for 2 h. Aliquots (100 μl) were spread plated on BHIA Cm10 containing 0.05% sodium citrate. Plates were incubated 37^o^C for 24h (pRAB11) or 28^o^C for 48h (pIMAY) and colony counts performed.

**References**

1. Lee JYH, Monk IR, Gonçalves da Silva A, Seemann T, Chua KYL, Kearns A, Hill R, Woodford N, Bartels MD, Strommenger B, Laurent F, Dodémont M, Deplano A, Patel R, Larsen AR, Korman TM, Stinear TP, Howden BP. 2018. Global spread of three multidrug-resistant lineages of *Staphylococcus epidermidis*. Nat Microbiol 3:1175-1185.
2. Seemann T. Snippy: Rapid bacterial SNP calling and core genome alignments. 2018. <https://github.com/tseemann/snippy.git>
3. Bankevich A, Nurk S, Antipov D, Gurevich AA, Dvorkin M, Kulikov AS, Lesin VM, Nikolenko SI, Pham S, Prjibelski AD, Pyshkin AV, Sirotkin AV, Vyahhi N, Tesler G, Alekseyev MA, Pevzner PA. 2012. SPAdes: a new genome assembly algorithm and its applications to single-cell sequencing. J Comput Biol 19:455–477.
4. Wick RR, Judd LM, Gorrie CL, Holt KE. 2017. Unicycler: Resolving bacterial genome assemblies from short and long sequencing reads. PLoS Comput Biol 13:e1005595.
5. Wood DE, Salzberg SL. 2014. Kraken: ultrafast metagenomic sequence classification using exact alignments. Genome Biol 15:R46.
6. Costa SK, Donegan NP, Corvaglia A-R, Francois P, Cheung AL. 2017. Bypassing the restriction system to improve transformation of *Staphylococcus epidermidis*. J Bacteriol 199:e00271–17.
7. Larsen MV, Cosentino S, Rasmussen S, Friis C, Hasman H, Marvig RL, Jelsbak L, Sicheritz-Ponten T, Ussery DW, Aarestrup FM, Lund O. 2012. Multilocus Sequence Typing of total-genome-sequenced bacteria. J Clin Microbiol 50:1355–1361.
8. Couvin D, Bernheim A, Toffano-Nioche C, Touchon M, Michalik J, Néron B, Rocha EPC, Vergnaud G, Gautheret D, Pourcel C. 2018. CRISPRCasFinder, an update of CRISRFinder, includes a portable version, enhanced performance and integrates search for Cas proteins. Nucleic Acids Res 46:W246–W251.
9. Monk IR, Howden BP, Seemann T, Stinear TP. 2017. Correspondence: Spontaneous secondary mutations confound analysis of the essential two-component system WalKR in *Staphylococcus aureus*. Nat Commun 8:ncomms14403.
10. Altschul SF, Gish W, Miller W, Myers EW, Lipman DJ. 1990. Basic local alignment search tool. J Mol Biol 215:403–410.
11. Sievers F, Wilm A, Dineen D, Gibson TJ, Karplus K, Li W, Lopez R, McWilliam H, Remmert M, Söding J, Thompson JD, Higgins DG. 2011. Fast, scalable generation of high-quality protein multiple sequence alignments using Clustal Omega. Mol Syst Biol 7:539–539.
12. Kearse M, Moir R, Wilson A, Stones-Havas S, Cheung M, Sturrock S, Buxton S, Cooper A, Markowitz S, Duran C, Thierer T, Ashton B, Meintjes P, Drummond A. 2012. Geneious Basic: an integrated and extendable desktop software platform for the organization and analysis of sequence data. Bioinformatics 28:1647–1649.
13. Monk IR, Tree JJ, Howden BP, Stinear TP, Foster TJ. 2015. Complete bypass of restriction rystems for major *Staphylococcus aureus* lineages. mBio 6:e00308–15.
14. Lee JYH, Monk IR, Pidot SJ, Singh S, Chua KYL, Seemann T, Stinear TP, Howden BP. 2016. Functional analysis of the first complete genome sequence of a multidrug resistant sequence type 2 *Staphylococcus epidermidis*. Microb Genom 2:e00007.
15. Datsenko KA, Wanner BL. 2000. One-step inactivation of chromosomal genes in *Escherichia coli* K-12 using PCR products. Proc Natl Acad Sci USA 97:6640–6645.
16. Monk IR, Shah IM, Xu M, Tan M-W, Foster TJ. 2012. Transforming the untransformable: application of direct transformation to manipulate genetically *Staphylococcus aureus* and *Staphylococcus epidermidis*. mBio 3, e00277-11.
17. Cherepanov PP, Wackernagel W. 1995. Gene disruption in *Escherichia coli*: TcR and KmR cassettes with the option of Flp-catalyzed excision of the antibiotic-resistance determinant. Gene 158:9-14.
18. Monk IR, Gahan CGM, Hill C. 2008. Tools for functional postgenomic analysis of *Listeria monocytogenes*. Appl Environ Microbiol:74;3921-3934.
19. Heckman KL, Pease LR. 2007. Gene splicing and mutagenesis by PCR-driven overlap extension. Nat Protoc 2:924-932.
20. Winstel V, Kühner P, Rohde H, Peschel A. 2016. Genetic engineering of untransformable coagulase-negative staphylococcal pathogens. Nat Protoc 11:949–959.
21. Foster TJ. 1998. 7.9 Molecular genetic analysis of staphylococcal virulence. Method Microbiol 27:433-454.
