# Supplementary material for: Mining the Methylome Reveals Extensive Diversity in Staphylococcus epidermidis Restriction Modification
Source: mBio. 2019 Dec 17;10(6):e02451-19. doi: 10.1128/mBio.02451-19 (PMC6918075; doi:10.1128/mBio.02451-19)
Supplement: TABLE S6 [file mBio.02451-19-st006.docx]

**Table S6. Strains, plasmids and oligonucleotides.**

| **Bacterial strain, plasmid or oligonucleotide** | **Description** | **Reference or Source** |
| --- | --- | --- |
| ***E. coli* strains** |  |  |
| DC10B | DH10B with ∆*dcm* mutation | (1) |
| Ec_Se662I  (previously DC10B-MS1) | DC10B with BPH0662 *hsdMS1* integrated between *ybbD* and *ylbG*. PB | (2) |
| Ec_Se662II  (previously DC10B-MS2) | DC10B with BPH0662 *hsdMS2* integrated between *essQ* and *cspB.* PB | (2) |
| Ec_Se662I-II | DC10B with BPH0662 *hsdMS1* integrated between *ybbD* and *ylbG*, BPH0662 *hsdMS2* integrated between *essQ* and *cspB* | (3) |
| Ec_Se736I | DC10B with BPH0736 *hsdMS* integrated between *essQ* and *cspB.* PB | This study |
| Ec_SeRP62aI | DC10B with RP62a *hsdMS* integrated between *gidB* and *atpI* | This study |
| ***S. aureus strains*** |  |  |
| PS187Δ*hsdR*Δ*sauPSI* | ST395 *S. aureus* deficient in type I and IV RM systems | (4) |
| ***S. epidermidis* strains** |  |  |
| 1457 | Clinical reference strain, isolated from infected central venous catheter; non-functional type I RM system; ST86 | (5,6) |
| ATCC 12228 | Non-clinical reference strain; non-functional type I RM system; ST8 | (7) |
| BPH0662 | Clinical reference strain, isolated from CSF; two functional type I RM systems ST2; PB  Cm10^S^, Tet5^S^, Ery30^R^, Kan50^R^ | (2) |
| BPH0662Δ*hsdSI* | Mutant of BPH0662 with *hsdS1*deletion | This study |
| BPH0662Δ*hsdSII* | Mutant of BPH0662 with *hsdS2* deletion | This study |
| BPH0662Δ*hsdSI*Δ*hsdSII* | Mutant of BPH0662 with both *hsdS1* and *hsdS2* deleted | This study |
| BPH0676 | Clinical strain, isolated from blood; no type I RM systems; ST2 | (3) |
| BPH0697 | Clinical strain, isolated from blood; one functional type I RM system; ST89; PB  Cm10^R^, Tet5^S^, Ery30^S^, Kan50^R^ | (3) |
| BPH0704 | Clinical strain, isolated from blood; one functional type I RM system; ST358; PB  Cm10^S^, Tet5^S^, Ery30^R^, Kan50^R^ | (3) |
| BPH0710 | Clinical strain, isolated from blood; non-functional type I RM system; ST2 | (3) |
| BPH0711 | Clinical strain, isolated from blood; one functional type I RM system; ST59. PB  Cm10^S^, Tet5^R^, Ery30^S^, Kan50^R^ | (3) |
| BPH0723 | Clinical strain, isolated from blood; one functional type I RM system; ST5. PB  Cm10^S^, Tet5^S^, Ery30^R^, Kan50^R^ | (3) |
| BPH0736 | Clinical strain, isolated from blood; non-functional type I RM system; ST2. PB  Cm10^S^, Tet5^S^, Ery30^R^, Kan50^S^ | (3) |
| BPH0736Δ*hsdS* | Mutant of BPH0736 with *hsdS* deletion | This study |
| BPH0747 | Clinical strain, isolated from blood; one functional type I RM system; ST2. PB  Cm10^R^, Tet5^S^, Ery30^S^, Kan50^R^ | (3) |
| RP62a_UoM | Clinical reference strain, isolated from blood; one functional type I RM system; ST10. PB  Cm10^S^, Tet5^S^, Ery30^R^, Kan50^R^ | (8,9) |
| RP62aΔ*hsdS* | Mutant of RP62a with *hsdS* deletion | This study |
| **Plasmids** |  |  |
| pKD4 | Plasmid for amplification of FRT-*kan*-FRT; Amp^R^, Kan^R^ | (10) |
| pKD46 | *E. coli* temperature sensitive plasmid containing λ red recombinase genes under the control of an arabinose-inducible promoter; Amp^R^ | (10) |
| pCP20 | *E. coli* temperature sensitive plasmid expressing *flp* enzyme for *flp*-catalyzed excision of *kan* marker; Amp^R^, Cm^R^ | (11) |
| pIMAY | Temperature sensitive, low copy number allelic exchange plasmid for staphylococci; Cm^R^ | (1) |
| pIMAY(∆662*hsdSI*) | pIMAY with BPH0662∆*hsdSI* construct in MCS | This study |
| pIMAY(∆662*hsdSII*) | pIMAY with BPH0662∆*hsdSII* construct in MCS | This study |
| pIMAY(∆736*hsdS*) | pIMAY with BPH0736∆*hsdS* construct in MCS | This study |
| pIMAY(∆RP62a*hsdS*) | pIMAY with RP62a∆*hsdS* construct in MCS | This study |
| pRAB11 | Non-temperature sensitive, high copy number staphylococci vector; Cm^R^ | (12) |
| **Phage** |  |  |
| Φ187 | *S. aureus* ST395 lineage specific phage | (13) |
| **Cloning *hsdMS* genes** |  |  |
| **Ec_Se736I** |  |  |
| IM199 (*essQ* tail + Pxyl/tetO promoter-736MS F) | CCCAAACTGCACCCAAGAGTCAGAACACAGTTTTTCAAGAGTACAAAGGGGTAAACTAAAATAAATATTGACACTCTATCATTG | (2) |
| 736MS-F (Pxyl/tetO promoter- 736MS F) | AAACTAAAATAAATATTGACACTCTATCATTGATAGAGTATAATTAAAATAAGGAGGAAATTAATGGCCACTATAGGTTTTGAAGAA | This study |
| IM201 (736MS R) | TTAAATTGAAAGTTCATCACTATTCACCTC | (2) |
| IM202 (pKD4 F) | GGTGAATAGTGATGAACTTTCAATTTAAGTGTAGGCTGGAGCTGCTTC | (2) |
| IM203 (pKD4 R + *cspB* tail) | GCTAACCATTGTGGTGAAGTGCAGGTTTGCTGCATGAATAGTTTTACGGTCCATATGAATATCCTCCTTAG | (2) |
|  |  |  |
| **Ec_SeRP62aI** |  |  |
| IM365 (RP62aMS F; NcoI/PstI site) | ATATCCATGGCAACGACGGAAAAACAAAG | This study |
| IM266 (RP62aMS R; NcoI/PstI site) | ATATCTGCAGTTACACAAACATTTTCTGTAGTAATCC | This study |
| IM367 (*aptI* tail + Phelp promoter-RP62aMS F) | CAAAAAGCGGTCAAATTATACGGTGCGCCCCCGTGATTTCAAACAATAAGTACGGAGCTCCATTATGCTTTGGCAG | This study |
| IM360 (*SOE to RP62aMS* + pKD4 F) | *CTACAGAAAATGTTTGTGTAACTGCAG*ATATGTGTAGGCTGGAGCTGCTTC | This study |
| IM366 (pKD4 R + *gidB* tail) | ATAACGTGGCTTTTTTTGGTAAGCAGAAAATAAGTCATTAGTGAAAATATGTCCATATGAATATCCTCCTTAG | This study |
|  |  |  |
| **∆*hsdS*** |  |  |
| BPH0662-A1 (pIMAY MCS/KpnI site) | CCTCACTAAAGGGAACAAAAGCTGGGTACCAGTGATATTGAAATTGATATGTTAGGG | This study |
| BPH0662-B1 | AAATCTTAATTCTGGAGTGTTTGTGTG | This study |
| BPH0662-C1 (*SOE to B*) | *CACACAAACACTCCAGAATTAAGATTT*TAGTTCTGATAACAGCCTATTATGTC | This study |
| BPH0662-D1 (pIMAY MCS) | CGACTCACTATAGGGCGAATTGGAGCTCGTGATTCTAGCTTATGGTGCTCAT | This study |
| BPH0662-A2 (pIMAY MCS/KpnI site) | CCTCACTAAAGGGAACAAAAGCTGGGTACCGGAGAATTCTATACGCCATCATC | This study |
| BPH0662-B2 | TCCATTAAACTCCATAACCTAGTCC | This study |
| BPH0662-C2 (*SOE to B*) | *GGACTAGGTTATGGAGTTTAATGGA*AATAGTGATGAACTTTCAATTTAATGAAG | This study |
| BPH0662-D2 (pIMAY MCS) | CGACTCACTATAGGGCGAATTGGAGCTCGACAACTAAGGTAGGATTATTAAGCATTTG | This study |
| BPH0736-A (pIMAY MCS/KpnI site) | CCTCACTAAAGGGAACAAAAGCTGGGTACCACAGCAGGATTCGTATTAGCC | This study |
| BPH0736-B | AGCTACATCACCGAAAAAAATTGT | This study |
| BPH0736-C (*SOE to B*) | *ACAATTTTTTTCGGTGATGTAGCT*ACAAATAGTAGACAAAGAGTAAAACCAACA | This study |
| BPH0736-D (pIMAY MCS) | CGACTCACTATAGGGCGAATTGGAGCTCCGATTGTCCTTCATCATCATAGTC | This study |
| RP62a-A (pIMAY MCS/KpnI site) | CCTCACTAAAGGGAACAAAAGCTGGGTACCAGGAGAATTCTATACACCACAACAG | This study |
| RP62a-B | AAATCTTAATTCTGGAGTATTTGTGTG | This study |
| RP62a-C (*SOE to B*) | *CACACAAATACTCCAGAATTAAGATTT*TAATTCTGATAAAGCCCTATTATGTTAATTG | This study |
| RP62a-D (pIMAY MCS) | CGACTCACTATAGGGCGAATTGGAGCTCCGACGCTATCACAACCTTACTTAAC | This study |
| **Recombineering** |  |  |
| IM179 (*essQ* out F) | CGGCCATTTATACAGGAAAAGCCTA | (2) |
| IM180 (*cspB* out R) | GTTACCTTCTCTATAGAGAGTGGTG | (2) |
| IM434 (*atpI* out F) | ACTTTCTTTAAGGCTTAGAGTCAAGC | This study |
| IM435 (*gidB* out R) | TTTAACGCCACGTTCACTCTTTTGC | This study |
| IM1 (pIMAY backbone F) | GGTACCCAGCTTTTGTTCCCTTTAGTGAGG | (3) |
| IM2 (pIMAY backbone R) | GAGCTCCAATTCGCCCTATAGTGAGTCG | (3) |
| IM3 (pIMAY MCS R) | AATACCTGTGACGGAAGATCACTTCG | (3) |
| IM4 (pIMAY MCS F) | TACATGTCAAGAATAAACTGCCAAAGC | (3) |
| BPH0662∆*hsdSI* out F | CGAAACTTATTTCGAAGGTCAT | This study |
| BPH0662∆*hsdSI* out R | GATAAACACACAGACATCCACTTG | This study |
| BPH0662∆*hsdSII* out F | AGATGTATTAGGCCGAGTGTATGAG | This study |
| BPH0662∆*hsdSII* out R | CCGTCCTTTCGATTTAACAAAC | This study |
| BPH0736∆*hsdSI* out F | ATGGCCACTATAGGTTTTGAAG | This study |
| BPH0736∆*hsdSI* out R | CACTACGATAGGCAAGCCATT | This study |
| RP62a∆*hsdSI* out F | AATTGATATGTTAGGGGATGCTTATG | This study |
| RP62a∆*hsdSI* out R | GGTTTTCAAGTGAATTTAAGGTGTG | This study |

PB = PacBio sequenced; RM = restriction modification; ST = sequenced type; CSF = cerebrospinal fluid; Cm10^R^ = chloramphenicol 10 μg/ml resistant; Cm10^S^ = chloramphenicol 10 μg/ml susceptible; Tet5 = tetracycline 5 μg/ml; Ery30 = erythromycin μg/ml; Kan50 = kanamycin 50 μg/ml; Amp = ampicillin; MCS = multiple cloning site; F = forward; R = reverse; SOE = spliced overlap extension.

**References**

1. Monk IR, Shah IM, Xu M, Tan M-W, Foster TJ. 2012. Transforming the untransformable: application of direct transformation to manipulate genetically *Staphylococcus aureus* and *Staphylococcus epidermidis*. mBio 3, e00277-11.
2. Lee JYH, Monk IR, Pidot SJ, Singh S, Chua KYL, Seemann T, Stinear TP, Howden BP. 2016. Functional analysis of the first complete genome sequence of a multidrug resistant sequence type 2 *Staphylococcus epidermidis*. Microb Genom 2:e00007.
3. Lee JYH, Monk IR, Gonçalves da Silva A, Seemann T, Chua KYL, Kearns A, Hill R, Woodford N, Bartels MD, Strommenger B, Laurent F, Dodémont M, Deplano A, Patel R, Larsen AR, Korman TM, Stinear TP, Howden BP. 2018. Global spread of three multidrug-resistant lineages of *Staphylococcus epidermidis*. Nat Microbiol 3:1175-1185.
4. Winstel V, Kühner P, Rohde H, Peschel A. 2016. Genetic engineering of untransformable coagulase-negative staphylococcal pathogens. Nat Protoc 11:949–959.
5. Mack D, Siemssen N, Laufs R. 1992. Parallel induction by glucose of adherence and a polysaccharide antigen specific for plastic-adherent *Staphylococcus epidermidis*: Evidence for functional relation to intercellular adhesion. Infect Immun 60:2048-2057.
6. Galac MR, Stam J, Maybank R, Hinkle M, Mack D, Rohde H, Roth AL, Fey PD. 2017. Complete genome sequence of *Staphylococcus epidermidis* 1457. Genome Announc 5:e00450-17.
7. Zhang YQ, Zhang YQ, Ren SX, Ren SX, Li HL, Li HL, Wang YX, Wang YX, Fu G, Fu G, Yang J, Qin ZQ, Miao YG, Wang WY, Chen RS, Shen Y, Chen Z, Yuan ZH, Zhao GP, Qu D, Danchin A, Wen YM. 2003. Genome‐based analysis of virulence genes in a non‐biofilm‐forming *Staphylococcus epidermidis* strain (ATCC 12228). Mol Microbiol 49:1577–1593.
8. Christensen GD, Bisno AL, Parisi JT, McLaughlin B, Hester MG, Luther RW. 1982. Nosocomial septicemia due to multiply antibiotic-resistant *Staphylococcus epidermidis*. Ann Intern 96:1-10.
9. Gill SR, Fouts DE, Archer GL, Mongodin EF, DeBoy RT, Ravel J, Paulsen IT, Kolonay JF, Brinkac L, Beanan M, Dodson RJ, Daugherty SC, Madupu R, Angiuoli SV, Durkin AS, Haft DH, Vamathevan J, Khouri H, Utterback T, Lee C, Dimitrov G, Jiang L, Qin H, Weidman J, Tran K, Kang K, Hance IR, Nelson KE, Fraser CM. 2005. Insights on evolution of virulence and resistance from the complete genome analysis of an early methicillin-resistant *Staphylococcus aureus* strain and a biofilm-producing methicillin-resistant *Staphylococcus epidermidis* strain. J Bacteriol 187:2426–2438.
10. Datsenko KA, Wanner BL. 2000. One-step inactivation of chromosomal genes in *Escherichia coli* K-12 using PCR products. Proc Natl Acad Sci USA 97:6640–6645.
11. Cherepanov PP, Wackernagel W. 1995. Gene disruption in *Escherichia coli*: TcR and KmR cassettes with the option of Flp-catalyzed excision of the antibiotic-resistance determinant. Gene 158:9-14.
12. Helle L, Kull M, Mayer S, Marincola G, Zelder M-E, Goerke C, Wolz C, Bertram R. 2011. Vectors for improved Tet repressor-dependent gradual gene induction or silencing in *Staphylococcus aureus*. Microbiol 157:3314–3323.
13. Winstel V, Kühner P, Krismer B, Peschel A, Rohde H. 2015. Transfer of plasmid DNA to clinical coagulase-negative staphylococcal pathogens by using a unique bacteriophage. Appl Environ Microbiol 81:2481–2488.
